# The assessment of qualitative olfactory dysfunction in COVID-19 patients: a systematic review of tools and their content validity

**DOI:** 10.3389/fpsyg.2023.1190994

**Published:** 2023-06-20

**Authors:** Annelin Espetvedt, Siri Wiig, Kai Victor Myrnes-Hansen, Kolbjørn Kallesten Brønnick

**Affiliations:** ^1^Department of Quality and Health Technology, Faculty of Health Sciences, University of Stavanger, Stavanger, Norway; ^2^SHARE–Centre for Resilience in Healthcare, Faculty of Health Sciences, University of Stavanger, Stavanger, Norway; ^3^The Cognitive and Behavioral Neuroscience Lab, University of Stavanger, Stavanger, Norway; ^4^The Norwegian School of Hotel Management, Faculty of Social Sciences, University of Stavanger, Stavanger, Norway

**Keywords:** assessment, qualitative olfactory dysfunction, tools, parosmia, phantosmia, COVID-19, content validity

## Abstract

**Background:**

There is a lack of overview of the tools used to assess qualitative olfactory dysfunction, including parosmia and phantosmia, following COVID-19 illness. This could have an impact on the diagnosis and treatment offered to patients. Additionally, the formulations of symptoms are inconsistent and often unclear, and consensus around the wording of questions and responses is needed.

**Aim of study:**

The aim of this systematic review is to provide an overview of tools used to assess qualitative olfactory dysfunction after COVID-19, in addition to addressing the content validity (i.e., item and response formulations) of these tools.

**Methods:**

MEDLINE, Web of Science, and EMBASE were searched 5^th^ of August 2022 and updated on the 25^th^ of April 2023 to identify studies that assess qualitative olfactory dysfunction in COVID-19 patients. Primary outcomes were the tool used (i.e., questionnaire or objective test) and item and response formulations. Secondary outcomes included psychometric properties, study design, and demographic variables.

**Results:**

The assessment of qualitative olfactory dysfunction is characterized by heterogeneity, inconsistency, and lack of validated tools to determine the presence and degree of symptoms. Several tools with overlapping and distinct features were identified in this review, of which some were thorough and detailed, while others were merely assessing the presence of symptoms as a binary measure. Item and response formulations are also inconsistent and often used interchangeably, which may lead to confusion, incorrect diagnoses, and inappropriate methods for solving the problem.

**Conclusions:**

There is an unmet need for a reliable and validated tool for assessing qualitative olfactory dysfunction, preferably one that also captures quantitative olfactory issues (i.e., loss of smell), to ensure time-effective and specific assessment of the ability to smell. A consensus around the formulation of items and response options is also important to increase the understanding of the problem, both for clinicians, researchers, and the patient, and ultimately to provide the appropriate diagnosis and treatment.

**Registration and protocol:**

The URL is https://www.crd.york.ac.uk/PROSPERO/display_record.php?RecordID=351621. A preregistered protocol was submitted and accepted (12.09.22) in the International prospective register of systematic reviews (PROSPERO) with the registration number CRD42022351621.

## 1. Introduction

During the past years of the COVID-19 pandemic there has been an increasing focus on the sense of smell. Besides symptoms like cough, fever and a sore throat, olfactory dysfunction soon became acknowledged as one of the main symptoms of COVID-19. This dysfunction not only involves the loss of smell, but also the change of it (Parma et al., 2020; von der Brelie et al., 2020). While quantitative symptoms involve the total or partial loss of smell, called anosmia and hyposmia, respectively, qualitative symptoms include parosmia, in which odors smell differently to what they usually do, and phantosmia, where odors are perceived in the absence of an odor source. Regarding parosmia, we refer to euosmia when odors are perceived as more pleasant than before, and cacosmia, where odors are experienced as less pleasant. In COVID-19 patients the occurrence of parosmia and phantosmia has been reported to be 23.4% (Lechien et al., 2023c) and 37% (Bousquet et al., 2022), respectively. The estimate varies, however, much because the temporal aspects vary across studies. That is, some measure the prevalence during the acute phase, while others assess persistent symptoms weeks or months following the initial infection (for a more detailed review, see Gary et al., 2023).

The olfactory epithelium is directly exposed to environmental factors in the surrounding air that may disrupt its functions. One such factor is the SARS-CoV-2 virus, known to cause COVID-19 illness. While the sustentacular cells have been suggested to be the target site for this virus, this theory is only confined to quantitative dysfunction (Butowt et al., 2022). When it comes to qualitative olfactory symptoms, on the other hand, two main theories have been proposed: the central theory holds that connections in the olfactory cortex are changed, while the peripheral theory explains parosmia as caused by changes in the olfactory epithelium, more specifically in the connection between olfactory sensory neurons and the glomeruli. These changes could lead to distorted signal processing in the olfactory cortex and distorted perception of odors (Parker et al., 2022a).

Experiencing a loss or change of the sense of smell can have a negative impact on appetite and nutritional status (Croy et al., 2014; Fjaeldstad and Smith, 2022; Otte et al., 2022), social relationships, personal hygiene, and safety, consequently affecting one's quality of life (Erskine and Philpott, 2020; Vaira et al., 2022). This emphasizes the importance of understanding the qualitative changes in olfaction, to provide appropriate care and treatment to those suffering from these symptoms.

However, in order to do so, olfactory changes must be measured and interpreted reliably and consistently. The methods and tools used to evaluate changes in smell appear heterogeneous across studies, which leads to a limited understanding of these symptoms. Some tools may provide detailed descriptions of the measured outcome, while in other cases one is only presented with a list of symptoms without any further explanations of what these involve. As such, content validity varies across tools, which may lead to confusion and misconception of information, both for the patient, clinician, and researcher. As a consequence, patients may be incorrectly diagnosed, and the care and treatment provided may also therefore be inadequate.

There is a need to systematically evaluate the assessment of olfactory changes and their content validity. To our knowledge, no previous review has been conducted to answer these research questions neither in the context of olfactory changes in general nor COVID-19. The primary aim of this review is to investigate what measurement tools (i.e., questionnaires and objective tests) are used in assessing qualitative olfactory alteration in COVID-19 patients, in addition to looking into their psychometric properties. The secondary aim is to assess content validity by investigating how tool items and response options are formulated.

## 2. Methods and design

This review follows the Preferred Reporting Items for Systematic Reviews and Meta-Analyses (PRISMA) reporting guideline (Page et al., 2021). However, we decided to modify the guidelines according to the applicability of the research questions, involving the exclusion of sections dealing with risk of bias (quality) assessment, effect measures, and data analyses, due to this not being relevant for the research questions investigated. We primarily wanted to provide an overview of the tools available and did not seek to assess the quality of the included studies. This would indeed be of interest and importance but was not a focus in this paper because of the nature of the research questions.

### 2.1. Databases and sources

The databases MEDLINE, Web of Science and EMBASE were searched the 5th of August 2022. The search was updated on the 25th of April 2023, with the exception of the EMBASE database, which at this time was inaccessible. Databases were chosen based on the topic of interest, being tied mainly to the biomedical and psychological sciences.

### 2.2. Eligibility criteria

Eligible reports had to include participants over the age of 18. However, studies assessing younger participants were also considered eligible as long as adults were included in the study. Participants of interest were those experiencing changes in smell following COVID-19 illness. Next, inclusion was restricted to quantitative, human studies available in full-text and written in English. We included published reports from 2019 to current date.

Excluded reports involved those including only participants under the age of 18, prior olfactory changes before COVID-19, any type of review, editorials, hypothesis articles, short communications, comments, correspondence, letters, abstracts only, posters, newspaper articles, book chapters, guidelines and proceedings of scientific meetings, animal studies, duplicates, pre-prints, articles not in English or full-text, and not peer-reviewed. Moreover, studies that did not assess smell separately from other chemosensory functions (e.g., taste) or that did not assess or report olfactory change separately from olfactory loss (e.g., assessing a general “olfactory dysfunction”) were excluded. This also applied to studies including specific patient groups (e.g., multiple sclerosis patients), due to how some conditions and illnesses may affect the sense of smell. Moreover, records were excluded when details were insufficient or not provided. For instance, reporting that “the presence of parosmia was noted” would not provide enough detail if the question(s) asked was not reported. After submitting the protocol in the International prospective register of systematic reviews (PROSPERO; registration number CRD42022351621), the eligibility criteria were discussed and edited as follows: initially, the criteria included PCR-confirmed COVID-19 illness. This was removed because the assessment of qualitative changes in smell does not depend on the test or method used to diagnose COVID-19. Furthermore, to ensure relevant studies were included, research notes (initially excluded) were also considered eligible. The changes were registered in the protocol in PROSPERO.

### 2.3. Search strategy

Based on the eligibility criteria, synonyms for each relevant term were searched and defined. Terms were added or removed from the search according to their contribution to results. After modifying the search, the final search strategy was as follows: (COVID-19 OR corona OR coronavirus OR SARS-CoV-2 OR “SARS-CoV-2” OR COVID-19 OR SARS-CoV-2) AND (“olfactory dysfunction” OR “olfactory disorder” OR “olfactory impairment” OR “olfactory change” OR “olfactory alteration” OR “change of smell” OR “smell change^*^” OR “altered smell” OR “smell alteration^*^” OR parosmia OR cacosmia OR phantosmia OR troposmia OR euosmia OR “qualitative olfact^*^” OR “qualitative smell^*^”). The full search strategy for each database can be found in Supplementary Data 1.

### 2.4. Study selection

#### 2.4.1. Exportation

All references were exported to Rayyan (Ouzzani et al., 2016), in separate reference files containing the results from MEDLINE, Web of Science and EMBASE, respectively.

#### 2.4.2. Duplicate detection

Duplicates were identified using the “detect duplicates” function in Rayyan and were assessed and deleted by one investigator (AE).

#### 2.4.3. Title and abstract screening

**Figure 1:**
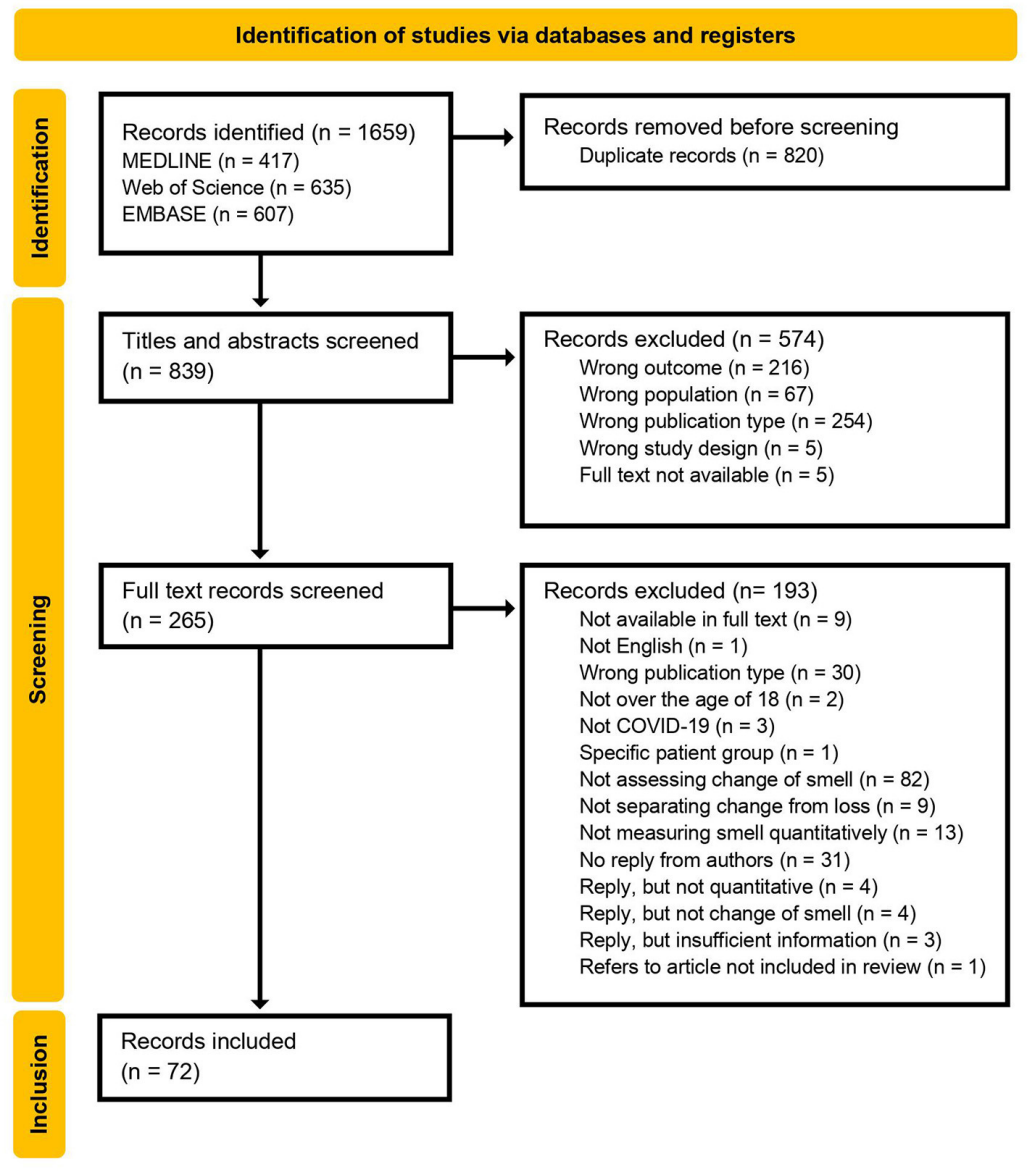
PRISMA flow diagram of included and excluded records, and exclusion reasons.

Title and abstract screening were performed in Rayyan, individually and blinded by two investigators (AE and KB). Records were included if they seemed potentially relevant. For instance, a record was included based on mentioning “olfactory dysfunction,” because it could potentially report the change of smell separately in the full text article. This applied unless definitions like “loss of smell” was used consistently throughout the abstract. Each record was evaluated manually and labeled *include* or *exclude*. If excluded, the record was labeled with a reason for exclusion. These reasons were later sorted into general categories (see Figure 1), where “wrong outcome” includes studies that did not look at the change of smell, for instance, “wrong population” includes studies done on animals or specific patient groups (like multiple sclerosis), “wrong publication type” includes article types like guidelines and systematic reviews, and “wrong study design” includes articles not assessing or reporting quantitative data, for instance.

#### 2.4.4. Full text screening

Full text screening was done according to the inclusion criteria, with focus on olfactory change and the tools used to assess this phenomenon. This process was done manually and individually by two blinded investigators (AE and KB) using Rayyan.

Records were labeled as included, excluded or maybe, in which the latter and conflicting results were discussed between the two investigators. When excluded, the reasons for this decision were noted in Rayyan, and can also be seen in the flow diagram (Figure 1).

When the tool was unavailable or not reported, the author of the report was contacted to obtain more information and preferably the full version. If authors did not reply within 2 weeks or if the means of measuring qualitative changes in smell were not quantitative, these records were excluded from the review.

#### 2.4.5. Data extraction

Data was collected from each report by noting all outcome variables in a table using MS Excel. A sample of ten reports were assessed individually and blinded by two investigators (AE and KB), followed by evaluating the correspondence between the extracted data. As consensus was satisfactory, the remaining reports were assessed individually by one investigator (AE).

Primary outcomes included the tool (e.g., questionnaire or objective test) used to assess change of smell, extracted in its full version, and the item and response formulations presented. If outcomes were not available in full text, authors were contacted for further information.

Secondary outcomes included the authors, year of publication, title of the report, phenomenon investigated (e.g., parosmia or phantosmia), type of tool (e.g., questionnaire or objective test), number of items, questions/statements verbatim, response design (i.e., the number of response alternatives and how these were structured and worded verbatim), estimated time to complete, scoring protocols (i.e., how scores were calculated), presence of normative data, original population target, reliability (i.e., internal consistency, test-retest reliability, and inter-rater reliability), validity (i.e., face validity, construct validity, criterion–including predictive and concurrent–validity, convergent validity, and discriminant validity), study type (e.g., cross-sectional), follow-up time, administration (e.g., online survey), language, setting (e.g., hospital), location (e.g., Spain), sample size, age, sex/gender, COVID severity (e.g., mild), and COVID stage (e.g., acute phase).

When there was no information about the validity or reliability of a tool (i.e., results of validation analyses), these secondary outcomes were considered not reported. Whenever the authors referred to a secondary reference for validation or reliability testing, this reference was sought for more details. For the sake of simplicity, when no information was provided regarding any test for validity or reliability, we report this collectively (e.g., “There was no information regarding validity of the tool”). This is, however, specified when such specific information was provided (e.g., “The tool had a good internal consistency”).

#### 2.4.6. Data synthesis

A narrative synthesis was done to analyse the extracted data. This was done individually by one investigator (AE). The synthesis involved summarizing and describing the outcome variables across studies. The results focus on the assessment of qualitative change of smell, that is, parosmia and phantosmia, and exclude parts not specific to this in the analysis. For instance, items concerning taste dysfunction were not analyzed.

## 3. Results

### 3.1. Study selection

#### 3.1.1. Exportation

All records (*n* = 1,659) were exported to Rayyan. According to each database result, MEDLINE identified 417 records, Web of Science identified 635 records, and EMBASE identified 607 records.

#### 3.1.2. Duplicate detection

During duplicate detection, 820 records were regarded as duplicates and removed. This was based on the manual assessment of the resemblance of the articles detected. This resulted in a total of 839 records eligible for title and abstract screening.

#### 3.1.3. Title and abstract screening

Two hundred sixty-five records were considered eligible for full text review, while 574 were excluded (see Figure 1 for exclusion reasons).

#### 3.1.4. Full text screening

Fifty-eight articles did not describe methods in full or include the full version of the assessment tools for qualitative change of smell, and so authors were contacted regarding details. Thirty-one of these did not respond and were excluded from the review. One of the records that failed to respond to the e-mail (Gupta et al., 2022b), was still included in the analysis because it included other tools for assessing qualitative olfactory dysfunction. Specifically, the study included questions about the presence of qualitative symptoms, but did not specify how these questions were worded. They study did, however, include another questionnaire that was sufficiently described, and so the article was included in this review. Of the 27 who did reply, 11 was excluded due to not being quantitative, not measuring change of smell, or providing insufficient information. Finally, 72 records were included for analysis.

The flow diagram (Figure 1) summarizes the full text screening process, included and excluded records, and the reasons for exclusion.

### 3.2. Study characteristics

The results are presented according to the type of tool (objective test or questionnaire) and to the specific tool used by the included studies. Regarding age, we decided to assess articles that also included subjects below the age of 18, as long as the study primarily assessed adults. Five reports included participants starting at the age of 12 (Vaira et al., 2022), 13 (Pendolino et al., 2022), and 15 (Qiu et al., 2020; Raad et al., 2021; Fjaeldstad et al., 2023) but only two of these reported the proportion of children included (6%; Qiu et al., 2020) and 5%; Raad et al., 2021). Detailed information of demographic variables and study design are summarized in Supplementary Tables 1, 2, respectively.

#### 3.2.1. Assessment tool properties

The properties of interest include number of items, questions/statements (reported verbatim), response design (reported verbatim), time to complete, scoring procedure, normative data, original population, reliability (e.g., internal consistency and inter-rater reliability), and validity (e.g., construct validity, convergent validity, and discriminant validity). The tools identified are presented below. For detailed accounts of each tool's properties and item and response formulations please see Supplementary Tables 3, 4, respectively.

##### 3.2.1.1. Objective tests

Four studies (Bussiere et al., 2021; Weiss et al., 2021; Hunter et al., 2023; Sekine et al., 2023) reported using objective tests for the assessment of qualitative olfactory dysfunction.

###### 3.2.1.1.1. Chemosensory perception test (CPT)

One of the included articles (Bussiere et al., 2021) reported developing and using this objective test in a cross-sectional study. The CPT was conducted via video consultations in English and French.

There was no information reported about the time estimated to complete the test or its reliability. For the validity assessment, authors reported sensitivity and specificity results, but only in relation to differentiating “normal olfactory function” (defined as a score above 30.5 on the Sniffin' Sticks test; Kobal et al., 1996) from “subjective olfactory dysfunction.” The authors did not specify whether such dysfunction included qualitative symptoms or not.

###### 3.2.1.1.2. Yale jiffy

Yale Jiffy is a test based on common household items, used in the online, English, longitudinal study by Weiss et al. (2021). It was originally developed for assessing smell loss in the general population. The questions are clear and specific to parosmia, and the survey takes 2 min to complete.

There was no information provided in the report regarding normative data, validity, or reliability.

###### 3.2.1.1.3. SCENTinel 1.1

SCENTinel 1.1 is self-administered and consists of four sub-tests including the ability to detect, identify, as well as rating the intensity and pleasantness of an odor. It was initially developed to discriminate subjects with anosmia from those with normal smell (Parma et al., 2021), and was further designed to assess subjects with qualitative symptoms (i.e., parosmia and/or phantosmia; Hunter et al., 2023). It takes < 5 min to complete.

The latter study, included in this review, aimed at validating the SCENTinel 1.1, and authors report an acceptable sensitivity of the test as a whole in discriminating qualitative symptoms both from quantitative olfactory dysfunction and normal sense of smell. When considering each sub-test, however, odor intensity was the only component that significantly distinguished qualitative symptoms from quantitative ones. All sub-tests were able to separate qualitative symptoms from normal sense of smell, however, with the exception of odor intensity.

###### 3.2.1.1.4. Sniffin' sticks parosmia test (SSParoT)

One study (Sekine et al., 2023) assessed the ability of the SSParoT (Liu et al., 2020) to distinguish patients with parosmia from those without such symptoms. Here, all patients had postviral olfactory dysfunction, including COVID-19 and other viral infections. The SSPAroT is based on the Sniffin' Sticks identification subtest, and measures hedonic distance between a pleasant and unpleasant odor, as well as the general perception of pleasant and unpleasant odors, resulting in two separate scores.

There was no information about the time it takes to complete the test. Regarding the validity of the SSParoT, it could distinguish healthy controls from patients with parosmia, but was not able to separate parosmia from non-parosmia.

##### 3.2.1.2. Questionnaires

The majority of studies reported using a questionnaire, either in its full, original version, or in an adapted form.

###### 3.2.1.2.1. National health and nutrition examination survey (NHANES)

Ten articles (Lechien et al., 2020a,b, 2021a,b, 2023a,b,c; Ninchritz-Becerra et al., 2021; Saussez et al., 2021; Alhazmi et al., 2022) reported using the NHANES, all of which used the 2011–2012 version (Centers for Disease Control Prevention, 2011). The NHANES includes a section on smell and taste, referred to as CSQ (Chemical senses–Taste and smell questionnaire). It was developed for the adult population, and was administered online in all the included studies, although consultations (i.e., seeing a physician to complete the assessment) were offered in two of these (Lechien et al., 2020a, 2021b) depending on the severity of the disease. The questions are clear and specific to the phenomena investigated. One of the studies (Alhazmi et al., 2022) had worded items differently as presented in the reports' results section but did not specify whether this was the format given to participants or only changed in the report.

None of the authors reported in what language the tool was given, but it has been administered in Italy, Spain, Belgium, France, USA, and Saudi-Arabia. There was no information in the reports regarding the time it takes to complete the questionnaire, normative data, validity, or reliability.

###### 3.2.1.2.2. Parosmia Questionnaire

Three of the included articles (Weiss et al., 2021; Boscolo-Rizzo et al., 2022a; Tuna and Tuna, 2023) reported using four questions originally developed by Landis et al. (2010). This set of items have not explicitly been named by the authors, apart from the running title in their original report. However, for the sake of order and clarity we will refer to them as the “Parosmia questionnaire” in this text. All items are formulated concisely, especially for the three latter questions. Question two and three are, however, biased in how it assumes that parosmia and phantosmia are limited to only involving unpleasant smells.

There was no information about the original population target, normative data, or estimated time to complete this questionnaire. Authors only refer to Landis et al. (2010) regarding validity and reliability. Here, Landis et al. report to what degree the four questions were suitable for distinguishing patients with parosmia from those without. In this regard, the gold standard for the presence or absence of parosmia was the result of the clinical judgement based on medical history during consultations. The fourth and first question were more specific and sensitive (surface under the curve = 0.83 and 0.76, respectively), compared to the second and third question (0.68 and 0.75, respectively).

###### 3.2.1.2.3. Questionnaire of olfactory disorders (QoD) and anglicized olfactory disorders questionnaire (eODQ)

Nine articles reported using the QoD, of which used the original German version developed by Frasnelli and Hummel (2005), and one used the English version (Chu et al., 2021).

The full German version consists of 29 questions, where four concerns parosmia, 17 are negative statements, two are positive statements, and six are investigating the degree to which answers are honest or socially desirable. Moreover, the QOD includes five visual analog scale questions about the impact on daily activities. Regarding the parosmic statements, these are not only asking about smell and not only about parosmia, but also about taste and phantosmia.

Regarding the eODQ, the four questions have been reduced and rephrased into two items. Like the questions in the Parosmia questionnaire, these items are also concise, but somewhat biased in terms of negative direction.

For both the QOD and the eODQ, there was no information regarding normative data or estimated time to complete.

QOD was reported to be validated (Liu et al., 2022; Otte et al., 2022; Prem et al., 2022), but no detailed information was provided. Instead, authors referred to Frasnelli and Hummel (2005) who reported significant correlations between negative statements and two tests for depression (*r* = 0.42) and mood (*r* = 0.46), as well as good internal consistency (α = 0.93) and test-retest reliability (*r* = 0.71). However, the type of validity testing was not specified, and it was unclear whether analyses included the parosmic statements or not. For the eODQ, this was reported to be validated (Chu et al., 2021), but details were not provided. Authors referred to Langstaff et al. (2019), who reported concurrent validity of *r* = −0.15 relative to the Sniffin' Sticks test (Hummel et al., 1997) as well as good internal consistency (α = 0.90).

###### 3.2.1.2.4. Global consortium for chemosensory research questionnaire (GCCR)

Five of the included studies used the GCCR questionnaire, of which two (Karni et al., 2021; Klein et al., 2021) report that the questionnaire they used was based on the GCCR. These questionnaires included all the GCCR questions about smell but also had two additional items that dealt with sense of smell and congested nose after illness. A third study (Bussiere et al., 2021) also based the questionnaire on the GCCR, but this was dissimilar in structure and content to such an extent that it was considered a self-developed questionnaire (discussed in detail below). The GCCR was developed by Parma et al. (2020) to measure smell, taste and chemesthesis function during and after recovery in patients with respiratory illness.

There was no information in the included articles regarding estimated time to complete, normative data, validity, or reliability.

###### 3.2.1.2.5. Olfactory dysfunction outcomes rating (ODOR)

Three of the included longitudinal online studies (Gupta et al., 2022b; Lee et al., 2022; Khan et al., 2023) used the ODOR, an English 28-item questionnaire. It is based on the QoD, of which two questions deal with phantosmia and parosmia. Items are worded in a clear, unambiguous manner.

The validity of the ODOR appears excellent, including internal consistency, test re-test reliability, concurrent, and discriminant validity (Lee et al., 2022), and only takes about 5 min to complete.

###### 3.2.1.2.6. Clinical global impression scale (CGI)

One of the aforementioned studies (Gupta et al., 2022b) also used the CGI in assessing the change of smell. This originally has a component for smell loss assessment, but researchers here asked about qualitative symptoms instead. This question is measuring smell dysfunction in general. At follow-up, however, improvement was assessed.

The follow-up question is vague in how it does not distinguish parosmia from phantosmia, and how it does not specify to the participant what the two concepts mean. Also, the item concerns the symptoms' impact in daily life, and not specifically the presence or severity of the symptoms.

The CGI used in the study was reported to be adapted from a validated outcome measure, but whether this adapted version was validated or tested for reliability was not specified. There was no information about the original population target, the time estimated to complete the questionnaire, or normative data.

##### 3.2.1.3. Self-developed questionnaires

The majority of included studies reported the use of a self-developed questionnaire that was used only for the purpose of the study. None of these reported any information regarding validity, reliability, completion time, or normative data. Only a few provided information about scoring procedures (Biadsee et al., 2020; Bussiere et al., 2021; Abo El Naga et al., 2022; Damiano et al., 2022).

One cross-sectional study (Bussiere et al., 2021) used an online questionnaire in English and French that was reported to be based on the GCCR, but the structure and wording of items differ to such a degree that it does not mirror the GCCR. This questionnaire also contains an objective smell test (CPT) previously described. Therefore, this questionnaire is considered a self-constructed, original tool in this review. It consists of 71 items, of which five concerns qualitative change of smell.

Sayin et al. (2021) reported that their questionnaire was based on the well-known Anosmia Reporting Tool (American Academy of Otolaryngology–Head Neck Surgery, 2021), but questions included were rephrased and authors also included new questions. Therefore, this questionnaire was also regarded as self-constructed in this review. It consists of 19 items, of which one concerned parosmia. Participants were presented with “Definition of smell impairment,” and were asked to choose “anosmia,” “hyposmia,” or “parosmia.” No further definition of the terms was provided, leading to a poor face validity. Authors did not specify in what language the questionnaire was given, but the cross-sectional study was carried out in Turkey.

Vaira et al. (2022) developed a 30-item questionnaire for their cross-sectional study, accessible in English and Italian in SurveyMonkey. Of these, four items were specific to parosmia and phantosmia.

In the cross-sectional study by Raad et al. (2021) the researchers designed an online questionnaire consisting of 27 items (although not numbered). The language of the questionnaire was not specified. Six items concerned the change of smell and six concerned rehabilitation and improvement of smell. Although the items are specific to the two symptoms, the response option that corresponds to parosmia could also be used to describe phantosmia. Moreover, the response option for phantosmia is biased in that it assumes that the odor perception is constant, which may not necessarily be the case.

Biadsee et al. (2020) developed an online 31-item questionnaire for their cross-sectional study. The language was not specified, but the study was carried out in Israel. Smell is assessed by five items, and qualitative smell dysfunction is evaluated by the following question: “Is your perception of smell distorted since COVID-19 onset, if yes describe?” The face validity is limited, as there was no definition in the questionnaire of what “distorted” implies.

Two studies by Chung et al. (2020, 2021) used a 17-item questionnaire (although questions are not numbered). The studies were carried out in China, but the language of the questionnaire was not specified. No clear definitions were provided in the questionnaire, leading to a poorer face validity.

In a longitudinal study by Lerner et al. (2022) researchers designed an English background questionnaire for assessing olfactory dysfunction online, and a follow-up version for those who reported parosmia in the first questionnaire. The questionnaires are not numbered, but contain several items about the distortion of smell, such as the onset date, temporal development, improvement, and medications or treatments. One question is “When you first noticed a change in your smell, did you experience a total loss of smell, decrease in smell, or distortion in smell?” There is no further information about what a distortion implies. As in the questionnaire by Raad et al. (2021), some of the next questions depend on this one response, in that they are worded in a non-specific manner, using the word “change.” Now, the follow-up questionnaire is more focused on the change of smell and defines parosmia at the very start of the questionnaire. While the baseline questionnaire has a somewhat limited face validity, since it does not specify what distortion implies, the follow-up questionnaire has a good face validity. However, it classifies phantosmia as part of the parosmia-definition.

Pendolino et al. (2022) used an English questionnaire developed for post-infectious olfactory dysfunction in their online, cross-sectional study. According to the report, it was developed in collaboration with AbScent, a United Kingdom charity that gathers people with smell disorders and has 18 items (although not numbered). Of these, two clearly present symptoms of parosmia and phantosmia. In the report, authors stated that the questionnaire had been validated by ear, nose, and throat clinicians and patient advocates, but there was no further information about the results of this process.

Another longitudinal study (Makaronidis et al., 2021) developed an online 18-item questionnaire, including follow-up symptoms assessed 4 to 6 weeks later. The wording of the question is concise, and the possibility for the participant to describe the issue also increases the validity of the item. The language of the tool was not specified, but Supplementary material was provided in English.

Gorzkowski et al. (2020) designed a 16-item telephone-based questionnaire in French and English for their cross-sectional study, of which two questions clearly and specifically assess parosmia and phantosmia. Authors reported that the questionnaire had been tested in 10 participants as part of a pilot study but had not been validated.

Ferdenzi et al. (2021) reported using a four-part French online questionnaire in their cross-sectional study. It was “… directed to people who noticed a change in their sense of smell…” and contains in total 14 items about chemosensory dysfunction. Two of these items deal with parosmia and phantosmia. Both questions are worded clearly and have the response format of “yes” and “no,” and the question about parosmia has an additional “describe.” This format was also used by Bousquet et al. (2022) in their study assessing phantosmia.

One of the more thorough parosmia-specific tools included in this review is the one designed by Parker et al. (2022b). This six-part online questionnaire measures parosmia in patients with COVID-19 and other etiologies (such as head trauma and viral infections). It was not specified in what language the questions were given, but the Supplementary material is in English. The questionnaire includes detailed assessment of potential triggers of parosmia, including 12 different foods, as well as unpleasant “bathroom smells.”

The assessment of various aspects was also included in other studies (Schwab and Fjaeldstad, 2022; Fjaeldstad et al., 2023). For instance, in the longitudinal study by Leung et al. (2022) researchers addressed the frequency, impact, and intensity of symptoms, and how these symptoms changed with time.

Hunter et al. (2023) presented participants with a question prior to the objective test, asking them to consider whether they had any quantitative or qualitative symptoms at the time. Here, descriptions of each symptom were worded in a clear manner. A similar approach was done in the study by Overdevest et al. (2022), however, questions also accounted for symptoms during COVID-19 illness as well as how often the symptoms were noticed by the participants.

Katsarou et al. (2022) developed a 14-item online questionnaire for their cross-sectional study, in which one of the items assessed the presence of parosmia: “Long COVID symptoms (please use a comma to separate each symptom).” Researchers analyzed the results by identifying term frequencies, of which parosmia was regarded as one of the categories. The question itself, however, allows for general and specific symptoms that participants themselves have to provide and define.

In addition to using the Parosmia questionnaire in one of their studies, Boscolo-Rizzo et al. (2022a,b) asked participants two questions: “Do you smell odors differently compared with previous experiences?,” and “Do you smell odors in the absence of an apparent source?” These questions are specific to symptom characteristics, limiting the degree of interpretation.

Patel et al. (2022) did a cross-sectional study in the US, where they asked participants one screening question during consultations: “Does anything smell different in the last year?,” followed by the response alternatives “yes” and “no.” Although the question describes parosmia, it allows for erroneous interpretation, as it does not directly exclude the experience of smelling odors that are not present or perceived by others.

A similar approach was used by Said et al. (2022), who developed an English online questionnaire where one question cross-sectionally assessed the presence of parosmia: “Do you currently have an altered sense of smell due to COVID-19 (aka parosmia)?.” Although authors provide the specific term parosmia in the question, the wording of the question does not directly define parosmia. As such, “altered sense of smell” is not confined to parosmia but could also include phantom smells or the loss of smell.

Polat et al. (2021) asked participants one question during longitudinal consultations: “How would you evaluate your ability to identify odors or taste compared to non COVID period of your life?” The responses were categorized by the researchers, one of which was termed parosmia. As in the aforementioned study, this question is not limited to parosmia, and may be biased toward participants responding with respect to quantitative issues, due to how it is worded “ability to identify….”

In two reports by Schambeck et al. (2021, 2022), researchers asked participants an open-ended question about olfactory dysfunction, to which several participants reported qualitative problems with smell. Consequently, authors developed a second questionnaire to assess these symptoms in more detail. In this questionnaire, the items are specific and defined in a non-ambiguous manner. There was no information in the report regarding the administration and language of this questionnaire.

Bhat et al. (2022) reported the results of an Indian case-study, based on a clinical consultation of one patient with parosmia. In the e-mail response, the corresponding author clarified that the patient would indicate the perceived discomfort and severity of parosmia on a visual analog scale. This way of scoring symptoms was also used in the study by De Luca et al. (2022) who evaluated the perception of 52 odors adapted from the Sniffin' Sticks test, and in the assessment of parosmia by Abo El Naga et al. (2022). There was no further information provided about whether a definition of parosmia was provided, and if so, how this symptom was described.

Di Stadio et al. (2022) reported that in their cross-sectional study, the participant was asked about parosmia and phantosmia during consultation with the question: “Did you perceive the odors differently after COVID-19 infection?,” followed by “if yes, did you smell bad odors? i.e., trash” and “if yes, did you perceive distorted odors?” The corresponding author specified in the e-mail response that these two latter questions assessed cacosmia and parosmia, respectively. Finally, they asked: “Could you score how much is altered the odor with a number from 0 (normal) to 10 (it is totally altered/unrecognizable.” The questions are specific to what they measure, but especially the first item assessing “bad odors” do not exclude that these bad odors may be perceived in the absence of an odor source, thus limiting the validity of results.

Several studies asked binary questions in a concise manner (Coelho et al., 2021; Callejón-Leblic et al., 2022; Damiano et al., 2022; Moideen et al., 2022; Turk et al., 2022). In other studies, however, questions were less clear or specific (Silverberg et al., 2022; Bérubé et al., 2023; Pendolino et al., 2023). For instance, Hosseininasab et al. (2021) asked participants about the presence of parosmia and phantosmia during longitudinal consultations and telephone interviews in Farsi. According to the corresponding author of the report, the researchers informed the participant that olfactory disorder was considered loss, reduction, or change in the sense of smell. The participant was then asked to indicate the onset and duration of anosmia, parosmia, and phantosmia. By asking about “change,” the question allows for interpretation and both general and specific responses but does not provide the participant with a definition of parosmia or phantosmia.

## 4. Discussion

### 4.1. Main findings

The aim of this review was to (1) identify the measurement tools (and their psychometric properties) used in assessing qualitative olfactory symptoms in COVID-19, and (2) investigate their content validity by investigating how items and response options were formulated.

We found a number of tools for assessing qualitative olfactory dysfunction, including a few objective tests, several standardized, widely used questionnaires, adaptations of these, and self-developed forms without any scoring protocol or assessment of statistical robustness. Most tools formulated items and response options in a concise manner, while some only partly did so or failed to specify the phenomena. The main findings are discussed in the order of tool types below.

#### 4.1.1. Objective tests

Only four objective tests were identified in this review, perhaps not surprisingly, considering how these qualitative symptoms are challenging to measure objectively. Moreover, the nature of the pandemic has made it difficult to develop, assess, and apply objective tests, as these may require meeting the participant face-to-face.

One of the studies excluded from this review due to a heterogeneous population sample (Gupta et al., 2022a) developed the Novel Anosmia Screening at Leisure (NASAL) test. The NASAL involves smelling seven items and reporting one's ability to smell the odor. Among the response categories is the option “smells different from normal,” which would indicate a qualitative issue with smell. It only takes about 10 min to complete, and its sensitivity and specificity has been evaluated. However, this was only with regards to distinguishing hyposmia and anosmia from normal olfactory function. Thus, whether it is also sensitive and specific to qualitative smell issues is unknown.

SCENTinel 1.1 is one of the tests that appears promising in distinguishing patients with parosmia from quantitative symptoms and normal sense of smell. However, when assessing sub-tests individually, odor intensity was the only component that significantly distinguished qualitative from quantitative symptoms. All sub-tests but odor intensity did, however, separate qualitative symptoms from normal sense of smell. Now, authors report including parosmia and/or phantosmia in the qualitative group, but report that phantosmia was not analyzed due to a low sample size.

Another objective test is the SSParoT, developed by Liu et al. (2020). In the initial development report, analyses suggested considerable agreement of the test-retest analysis, but the study only included three patients, of whom only one had phantosmia. Moreover, only participants under the age of 35 were included. In the article we included in this review (Sekine et al., 2023), the hedonic distance subtest was 29% sensitive and 67% specific to distinguishing healthy controls from those with parosmia. However, there was no difference between patients with and without parosmia.

These tests may be useful in the assessment of parosmia. However, parosmia is not only characterized by a hedonic aspect, but also the change in odor character (that is–not smelling like the item it usually smells like). Moreover, it is questionable how specific the tests are to phantosmia. Neither the SCENTinel 1.1 nor the SSParoT included phantosmia in the analyses, leading to unanswered questions regarding their ability to capture this phenomenon.

Objective tests would be of great value in providing reliable and consistent data. However, these need to be assessed regarding validity specific to qualitative olfactory dysfunction, which at this point seems to be limited. These should also extend to assess phantosmia, as this symptom seems not to have been included in any of the objective tests identified. However, this may be due to the difficulty of designing such a test. One could not simply present an odor stimulus and measure the presence of phantom smells, because phantosmia occurs in the absence of odor stimuli. Now, phantosmia could be triggered by other stimuli, such as sounds, visual input, or environmental factors, and although it may be challenging to minimize confounding factors in such a test, it would be interesting to explore this further.

#### 4.1.2. Questionnaires

Several tools with overlapping and distinct features were identified in this review, of which some were thorough and detailed, while others were merely assessing the presence of symptoms as a binary measure.

The most widely used questionnaires identified were the NHANES, QoD, and GCCR. These were all covering olfactory dysfunction, with the inclusion of a few questions about qualitative symptoms. One of our main interests is the validity of items assessing qualitative symptoms, but such details have been difficult to obtain or only presented vaguely.

The QoD is perhaps one of the most feasible and short questionnaires in assessing qualitative olfactory dysfunction. However, the primary aim of the tool is to assess quality of life related to olfactory dysfunction, and not symptoms *per se*. Another issue of interest is the formulation of the fourth question. By using the word “accident,” this item is somewhat biased and restricted, and would perhaps be regarded as irrelevant for some patients. In the context of COVID-19, it is reasonable to assume that participants may not consider the illness an accident as such, potentially affecting how or if they respond to the question. Also, there are only four questions specific to qualitative issues, of which only one of these addresses phantosmia. Now, interestingly the scale is only referred to as “parosmic,” even though it contains the question about phantom smells. This leads to the question: Did developers of this tool consider phantosmia to be part of parosmia as one concept, or was phantosmia left out of the assessment procedure? None of the included relevant studies specified what phenomena were measured, and so it could be that phantosmia was not assessed in participants. Another possibility is that it was assessed, but not reported. Although the items in the scale are worded in a non-ambiguous manner, this highlights the importance of providing clear definitions, not only to the participants receiving the questions, but also to clinicians or researchers that are to interpret the results and classify the symptoms observed.

In addition, the questions are biased toward a negative direction, leaving no room for symptoms characterized by perceiving pleasant odors, either in the presence or absence of an odor source. Although the majority of participants with qualitative smell issues may experience unpleasant odors, this may not necessarily be the case for every participant. However, when items are biased toward “unpleasant,” these cases are consequently excluded from the data collected. The questionnaire by Parker et al. (2022b) is the only questionnaire that directly measured potential euosmia, the positive aspect of parosmia, although other studies have included free-text entries where participants could describe such experiences. Euosmia has interestingly gained little or no attention in the assessment of qualitative olfactory dysfunction. This may be due to its positive character and how it may not have an impact on the quality of life, as opposed to cacosmia, where pleasant or neutral odors change into smelling foul. Nonetheless, it is still a symptom which may be of importance in evaluating the sense of smell. For instance, if a person can no longer perceive an odor as before, and the ability to quantitatively identify an odor is tested, the person may fail in identifying the odor. However, it may not be the identification itself that is the problem, but the altered perception of that specific odor. Consequently, this positive change of smell may also impact how quantitative olfactory function is scored and labeled.

Other questionnaires identified include the Parosmia questionnaire, ODOR, and CGI. While the ODOR is a validated and time-efficient questionnaire, its focus is on the quality of life relative to olfactory symptoms. Hence, the evaluation of parosmia and phantosmia as symptoms are not given much attention. The Parosmia questionnaire is essentially identical to the QoD, but differs in the wording of items, response design, and scoring procedure. As in the QoD, items are formulated in a negative direction (with focus on unpleasant odors), and there is one item assessing phantom smells. This item was found to have the least specificity and sensitivity compared to the other items (Landis et al., 2010), which leads to questioning the ability of the tool in capturing this symptom in addition to parosmia. On that note, one may also ask why the scale's name is restricted to parosmia when it also assesses phantosmia. The CGI provided no definition of parosmia and phantosmia to the participants, and the tool only asked about “… parosmia or phantosmia.” In this sense, it was not specific to the type of qualitative issue.

Most studies used a self-developed questionnaire, and failed to report validity, reliability, completion time, normative data, and-apart from a few-scoring procedures. A few were based on established tools, such as the GCCR, but were considered self-developed due to substantial differences. Several of the questionnaires were numerous in items, but only contained a few questions about qualitative smell issues. For instance, Bussiere et al. (2021) asked participants 71 questions, although only one assessed the presence of qualitative symptoms and four were part of the CPT. Also the validated ODOR includes only two items about qualitative issues. This may lead to asking the question: How validly and reliably can one or two questions capture distinct symptoms of qualitative olfactory dysfunction? And does it suffice only stating the presence of such symptoms, or is it necessary to understand aspects like duration and triggers to adequately diagnose and treat these issues? It could be that one item alone can capture the presence of a symptom. However, it seems impossible to capture aspects like duration, intensity, and triggering factors. These are important for understanding the phenomena but will not be addressed by the mere presence of symptoms. Of course, the inclusion of such aspects depends on the aim of the research, but we regard this inclusion as reasonable as it would improve judgements related to diagnosis and treatment.

Two of the more thorough questionnaires include the one designed by Lerner et al. (2022) and Parker et al. (2022b). The former included a baseline and follow-up questionnaire, where the baseline questionnaire had a somewhat limited face validity with regards to using the word “distortion.” Although one can assume that it involves parosmia, the word does not explicitly exclude phantosmia as a symptom. The follow-up questionnaire, on the other hand, provided a well-defined definition of parosmia at the very start of the form. Also, the title “Parosmia Follow up” aid in specifying what is being measured. Interestingly, one of the sections in this follow-up questionnaire asks about smell distortions, to which participants can select options like “burning smell” and “rotten meat,” but also the option “phantom smells.” Although this latter option is clearly defined, the inclusion of this symptom as part of the parosmia concept does question the clarity of what is in fact being measured.

While several questionnaires were clear and concise, some presented questions with less clarity or no explanation at all. Interestingly, in the questionnaire by Chung et al. (2021) researchers presented both “Parosmia” and “Cacosmia” without defining what these symptoms involve and how they differ from one another. The question was designed in such a way that participants could only provide true answers if they were already aware of their issues with smell, the meaning of the terms, and the differences between these. For a participant not familiar with these terms, providing a correct response may be difficult. Consequently, the inferences drawn from these results may not reflect the true prevalence of symptoms.

A similar issue concerns the use of terms like “change” or “distorted” and the lack of specifying what these terms mean. Although the questions asked may specify odors as “distorted,” none of these questions were worded in a manner that explicitly excluded phantosmia as a symptom. As such, participants may consider their phantom smells part of the parosmia concept. The result may be the presentation of a higher incidence of parosmia relative to the true prevalence. What is more, “change” could also be interpreted as a weaker sense of smell. This is not a qualitative issue, and so interpreting it as one could lead to falsely defining a symptom as qualitative when it is in fact reflective of smell loss.

While some questionnaires were structured in sections and numbered, others relied on one or two questions asked during consultation, or a single free-text entry that were later sorted into categories by the researchers. Although this latter format gives space for the inclusion of symptoms that do not fit into pre-fixed categories, it could also lead to missing information that could be collected had the question been specific and defined. As such, participants without knowledge of odor distortions or phantom smells, or who did not consider these symptoms to be abnormal, would perhaps not think to note these as COVID-related symptoms.

The heterogeneity of tools used to assess qualitative olfactory function is substantial. In addition, many of the reports lack transparency, clear methodological procedures, and clarity in item and response formulations. Many studies relied on clinical assessment and medical records in assessing symptoms (e.g., Hosseininasab et al., 2021; Moideen et al., 2022), and it was unclear whether clinicians used specific tools in this process. This challenges the degree of consistency and accuracy of results, makes it difficult to replicate methods, and by this limits the degree to which we can draw conclusions across studies.

### 4.2. Strengths and weaknesses

This review casts light over the tools used to assess change of smell following COVID-19 and provide an up-to-date overview which has previously been lacking. What is more, it also reflects heterogeneity and at times a poor content validity of the tools identified.

Regarding psychometric properties, most tools appear not validated or tested with regards to reliability. However, information was only sought in secondary articles whenever a validation process was reported in the included article. If several sources were sought, it may have resulted in more detailed information. However, this process would have extended beyond the scope, inclusion criteria, and time restrictions of this review, and the external search was limited to the sources provided in the included articles only.

On that note, there are likely additional tools existing outside the scope of this review, as olfactory dysfunction, both loss and change of smell, occurs not only in COVID-19 but also in a range of other populations. These tools have not been considered for the sake of feasibility and specificity.

### 4.3. Implications for future research

This review has revealed the following knowledge gaps: First, there is a lack of validated and reliable tools for measuring the change of smell in COVID-19, but presumably also other patient groups and populations with qualitative olfactory symptoms. Second, item and response formulations are found to be inconsistently defined and presented. Focus should be paid toward providing clear, unambiguous, and defined items and response options, that differ in a lesser degree across studies. By improving content validity, we can more readily identify the symptoms and provide the care appropriate for the issue in question. These findings motivate the development of a novel tool for measuring both the loss and change of smell separately, which will be the objective of a future study. Here, we will include both parosmia and phantosmia, and pilot and validate the novel questionnaire in Norwegian and English as part of an observational study. In line with the findings, it would be preferrable to develop an objective tool, but due to restricted time and resources, this would not be feasible. When it comes to phantosmia as a phenomenon, this seems challenging to capture, especially objectively. Also, we believe that a questionnaire could be a time-efficient, low-cost alternative that may give both the clinician and the patient insight into the problem that would perhaps not be possible using an objective test.

### 4.4. Conclusion

The assessment of qualitative olfactory dysfunction in COVID-19 is characterized by heterogeneity, inconsistency, and lack of validated tools to determine the presence and degree of symptoms. A great number of tools with overlapping and distinct features were identified in this review, of which some were thorough and detailed, while others were only noting symptoms as present or not. Although a few objective tests appear promising in the assessment of parosmia, research is limited regarding whether they also can identify phantosmia. This highlights the need for a reliable and validated tool for assessing both parosmia and phantosmia, preferably one that also separately captures the loss of smell. This could prove to be a time-effective and specific method of assessing the ability to smell. Regarding the content validity of tools, items and responses differ in their degree of clarity, consistency, and specificity. A consensus around how symptoms are measured is important to increase the understanding of the problem, both for clinicians, researchers, and the patient, and ultimately to provide the appropriate diagnosis and treatment.

## Data availability statement

The original contributions presented in the study are included in the article/Supplementary material, further inquiries can be directed to the corresponding author.

## Author contributions

The data extraction and writing of the article and Supplementary material were mainly done by AE. KB contributed to developing the search strategy, selecting studies, and extracting data. All authors contributed to revision and approval of the submitted manuscript.
