# Supplementary material for: The assessment of qualitative olfactory dysfunction in COVID-19 patients: a systematic review of tools and their content validity
Source: Front Psychol. 2023 Jun 20;14:1190994. doi: 10.3389/fpsyg.2023.1190994 (PMC10319418; doi:10.3389/fpsyg.2023.1190994)
Supplement: Supplementary file 1 [file Data_Sheet_1.docx]

Supplementary Material

The assessment of qualitative olfactory alteration in COVID-19 patients: A systematic review of tools and their content validity

Annelin Espetvedt*, Siri Wiig, Kai Victor Myrnes-Hansen, Kolbjørn K. Brønnick

*** Correspondence:** Annelin Espetvedt: annelin.espetvedt@uis.no

# Supplementary Data

# Supplementary data includes a summary of the search strategy (Supplementary Data 1), and four tables of demographic variables, study design, tool properties, and item and response formulations (Supplementary Tables 1, 2, 3, and 4, respectively).

# Supplementary Data 1. Full search strategy in MEDLINE, Web of Science and EMBASE.

MEDLINE (Ebsco):

(Covid19 OR corona OR coronavirus OR sars-cov-2 OR “sars cov 2” OR covid-19 OR sarscov2) AND ("olfactory dysfunction" OR "olfactory disorder" OR "olfactory impairment" OR "olfactory change" OR "olfactory alteration" OR "change of smell" OR "smell change*" OR "altered smell" OR "smell alteration*" OR parosmia OR cacosmia OR phantosmia OR troposmia OR euosmia OR "qualitative olfact*" OR "qualitative smell*")

Limited to: *Scholarly (Peer Reviewed) Journals; English Language; Human; from 2019-2023.*

Web of Science:

(Covid19 OR corona OR coronavirus OR sars-cov-2 OR “sars cov 2” OR covid-19 OR sarscov2) AND ("olfactory dysfunction" OR "olfactory disorder" OR "olfactory impairment" OR "olfactory change" OR "olfactory alteration" OR "change of smell" OR "smell change*" OR "altered smell" OR "smell alteration*" OR parosmia OR cacosmia OR phantosmia OR troposmia OR euosmia OR "qualitative olfact*" OR "qualitative smell*")

Limited to: *English Language; from 2019-2023.*

EMBASE:

(Covid19 OR corona OR coronavirus OR sars-cov-2 OR “sars cov 2” OR covid-19 OR sarscov2") AND ("olfactory dysfunction" OR "olfactory disorder" OR "olfactory impairment" OR "olfactory change" OR "olfactory alteration" OR "change of smell" OR "smell change*" OR "altered smell" OR "smell alteration*" OR parosmia OR cacosmia OR phantosmia OR troposmia OR euosmia OR "qualitative olfact*" OR "qualitative smell*")

Limited to: *English Language; from 2019–2023; human.*

# Supplementary Tables

## Supplementary Table 1. Individual studies’ demographic data.

| Year | 1^st^ author | | Title | Sample size | Age | Sex | C-19 severity | C-19 phase |
| --- | --- | --- | --- | --- | --- | --- | --- | --- |
| 2021 | Lechien | Epidemiological, otolaryngological, olfactory and gustatory outcomes according to the severity of COVID-19: a study of 2579 patients | | 2579 | Mean 44.4 (SD 16.7) | 1630 (63.2%) | Mild, moderate, and severe-to-critical | NR |
| 2020 | Lechien | Olfactory and gustatory dysfunctions as a clinical presentation of mild-to-moderate forms of the coronavirus disease (COVID-19): a multicenter European study | | 417 | Mean 36.9 (SD 11.4, range 19-77) | 263 (63.1%) | Mild-to-moderate | Only report 34.5% acute, otherwise not specified |
| 2021 | Saussez | Short-Term Efficacy and Safety of Oral and Nasal Corticosteroids in COVID-19 Patients with Olfactory Dysfunction: A European Multicenter Study | | 152 | Only reported for each of three groups with olfactory dysfunction: Mean 37.1 (SD 11.9), 42.95 (SD 12.66), 43.5 (SD 14.25) | Only reported for each of three groups with olfactory dysfunction: 35 (59.3%), 15 (68.2%), 40 (56.4%). | Mild | NR |
| 2021 | Ninchritz-Becerra | Subjective evaluation of smell and taste dysfunction in patients with mild COVID-19 in Spain | | 1043 | Mean 39 (SD 12, range 19-78) | 663 (63.6%) | Mild-to-moderate | Only report 42% acute, otherwise not specified |
| 2021 | Lechien | Prevalence and 6-month recovery of olfactory dysfunction: a multicentre study of 1363 COVID-19 patients | | 2581 (1369 completed follow-up) | Mean 44.5 (SD 16.4) | 1624 (62.9%) | Mild, moderate, severe, and critical | Only report acute and assessment 2 months after COVID-19 infection |
| 2020 | Lechien | Objective olfactory evaluation of self-reported loss of smell in a case series of 86 COVID-19 patients | | 86 | Mean 42 (SD 12) | 56 (65.1%) | Mild-to-moderate | NR, only report participants with symptoms for less and more than 14 days |
| 2022 | AlHazmi | COVID-19 infection related olfactory dysfunction in Saudi Arabia: Community-based study | | 392 | Only report age in intervals: 18-25 (111, 63.8%), 26-35 (35, 20.1%), 36-45 (13, 7.5%), 46-55 (12, 6.9%), and >55 (3, 1.7%) | 130 (74.7%) | NR | NR, only report assessing whether participants are recovered or still in acute phase |
| 2022 | Boscolo-Rizzo | Parosmia assessment with structured questions and its functional impact in patients with long-term COVID-19-related olfactory dysfunction | | 98 | No mean reported, only frequencies of three age groups: <40 (33), 40-49 (23), >50 (42) | 68 (69.4%) | NR | NR |
| 2021 | Weiss | Tracking smell loss to identify healthcare workers with SARS-CoV-2 infection | | 473 | Median 30 (IQR: 26-48; positive), 34.5 (IQR 29-44; negative) | 15 (88%; positive), 358 (79%; negative) | NR | NR |
| 2022 | Otte | Impact of COVID-19-Mediated Olfactory Loss on Quality of Life | | 65 (patients), 32 (healthy controls), 14 (controls with other aetiology) | Mean 44.9 (SD 11.7; patients), 51.8 (SD 15; healthy controls), 58.6 (SD 11.32; controls with other aetiology) | 41 (63%, patients), 19 (59%, healthy controls), 12 (85%, controls with other aetiology) | Only refer to "outpatient" | NR, only report that participants had recovered for at least 2 months |
| 2020 | Qiu | Olfactory and Gustatory Dysfunction as an Early Identifier of COVID-19 in Adults and Children: An International Multicenter Study | | 161 | 38.8 (IQR 23-53) | 69 (43%) | Asymptomatic, mild, moderate, severe, and critical | NR |
| 2022 | Prem | Long-lasting olfactory dysfunction in COVID-19 patients | | 102 | Mean 38.8 (SD 13.2, range 18-68) | 71 (69.6%) | NR | NR |
| 2021 | Liu | Olfactory-related Quality of Life Adjustments in Smell Loss during the Coronavirus-19 Pandemic | | 149 | Mean 47.5 (SD 47.5±15.7) | 71% | NR | NR, only report assessing new-onset and persistent smell loss (>30 days) |
| 2021 | Chu | Implementing a COVID-19 specialist smell clinic: experience at the Wrightington, Wigan and Leigh Teaching Hospitals (WWL), NHS Foundation Trust, United Kingdom | | 16 | Median 35 (range 20-55) | 13 (81%) | NR | NR, only report participants with olfactory impairment for at least 3 months |
| 2021 | Karni | Self-Rated Smell Ability Enables Highly Specific Predictors of COVID-19 Status: A Case-Control Study in Israel | | 112 (positive) and 112 (negatives) | Mean 35 (SD 12; positive COVID group), 37 (SD 12; negative COVID group) | (36%; positive), (52%; negative) | Only refer to "hospitalised" and "ambulatory" | NR |
| 2021 | Klein | Onset, duration and unresolved symptoms, including smell and taste changes, in mild COVID-19 infection: a cohort study in Israeli patients | | 103 | Mean 35 (SD 12) | (36%) | Mild-to-moderate | NR, only report follow-up for 6 months |
| 2020 | Parma | More than smell - COVID-19 is associated with severe impairment of smell, taste, and chemesthesis | | 4039 | Mean 41.38 (SD 12.20, range 19-79) | 2913 (72%) | NR | NR |
| 2022 | Ohla | A follow-up on quantitative and qualitative olfactory dysfunction and other symptoms in patients recovering from COVID-19 smell loss | | 1468 | Mean 43.89 (SD 12.17) | 1111 (75.68%) | NR | NR |
| 2022 | Delgado-Losada | Long-Lasting Olfactory Dysfunction in Hospital Workers Due to COVID-19: Prevalence, Clinical Characteristics, and Most Affected Odorants | | 164 | Mean 48.3 (SD 11.7; no COVID group), 43.3 (SD 14.6; asymptomatic group), 44.7 (SD 13; symptomatic group) | 62 (81.5%; no COVID group), 24 (82.7%; asymptomatic group), 49 (83%; symptomatic group) | NR | NR, only report that PCR test was confirmed at least 10 weeks ago |
| 2021 | Bussière | Chemosensory Dysfunctions Induced by COVID-19 Can Persist up to 7 Months: A Study of Over 700 Healthcare Workers | | 704 | Mean 42 (SD 11.7, range 18-70) | 593 (84.2%) | NR | NR, only report assessing participants 3-7 months after COVID-19 infection |
| 2022 | Vaira | The Effects of Persistent Olfactory and Gustatory Dysfunctions on Quality of Life in Long-COVID-19 Patients | | 431 | Mean 38.4 (SD 12.5, range 12-71) | 329 (76.3%) | NR | Acute and post-acute |
| 2020 | Biadsee | Olfactory and Oral Manifestations of COVID-19: Sex-Related Symptoms-A Potential Pathway to Early Diagnosis | | 128 | Mean 36.25 (range 18-73) | 70 (54.6%) | Mild | NR |
| 2021 | Sayin | Taste and Smell Impairment in COVID-19: An AAO-HNS Anosmia Reporting Tool-Based Comparative Study | | 128 | Mean 38.63 (SD 10.08, range 21-77) | 80 (62.5%) | Critically ill | NR |
| 2021 | Chung | Neurosensory rehabilitation and olfactory network recovery in COVID-19-related olfactory dysfunction | | 2 (and 2 controls) | 19, 41 (patients) 26, 29 (controls) | 50 % | Mild | NR |
| 2020 | Chung | Olfactory Dysfunction in Coronavirus Disease 2019 Patients: Observational Cohort Study and Systematic Review | | 36 (18 COVID and 18 controls) | Median 28 (SD 19; patients), 31 (SD 17.5; controls), (IQR 18-59) | 11 (61%, patients), 13 (72%, controls) | Mild | NR |
| 2022 | Lerner | Clinical Features of Parosmia Associated With COVID-19 Infection | | 148 (from a total of 231, where 83 did not have parosmia) | Mean 40.2 (variance not reported) | 78 (69.6%) | NR | NR |
| 2022 | Pendolino | The Role of Social Media in Improving Patient Recruitment for Research Studies on Persistent Post-Infectious Olfactory Dysfunction | | 65 | Median 55 (IQR 46-63, range 13-76) | 47 (72%) | NR | NR, only report that olfactory impairment must have been present for at least 12 months |
| 2022 | Gupta | Efficacy and Safety of Saline Nasal Irrigation Plus Theophylline for Treatment of COVID-19-Related Olfactory Dysfunction: The SCENT2 Phase 2 Randomized Clinical Trial | | 51 | 46 (SD 13.1) | 36 (71%) | NR | Post-COVID |
| 2021 | Makaronidis | Distorted chemosensory perception and female sex associate with persistent smell and/or taste loss in people with SARS-CoV-2 antibodies: a community based cohort study investigating clinical course and resolution of acute smell and/or taste loss in people with and without SARS-CoV-2 antibodies in London, UK | | 467 (381 positive and 86 negative) | Mean 39.67 (SD 12.12; positive), 40.25 (SD 12.33; negative) | 270 (70.9%; positive), 57 (66.3%; negative) | NR | Acute |
| 2020 | Gorzkowski | Evolution of Olfactory Disorders in COVID-19 Patients | | 229 | Mean 39.7 (SD 13.7; range 18-89) | 147 (64%) | Only refer to "outpatients" and "hospitalised" | NR |
| 2021 | Ferdenzi | Recovery from COVID-19-Related Olfactory Disorders and Quality of Life: Insights from an Observational Online Study | | 3111 | Mean 40.5 (SD 12.5, range 18-85) | 2429 (78.1%) | NR | NR |
| 2022 | Parker | Emerging Pattern of Post-COVID-19 Parosmia and Its Effect on Food Perception | | 727 (of which 606 had COVID-19) | Mean 43 (range 18-75) | 651 (90%) | Loss of smell only, mild, moderate, severe, and very severe (hospitalised) | Post-COVD (although time from infection is not specified) |
| 2021 | Raad | Parosmia in patients with COVID-19 and olfactory dysfunction | | 1299 | Only report age in intervals: 15-20: 7 (5%), 12-30: 52 (37.1%), 31-40: 57 (40.8%), 41-50: 22 (15.7%), 51-60: 2 (1.4%), >60: 0 (0%) | 103 (73.6%) | NR | NR |
| 2022 | Katsarou | The Greek Collaborative Long COVID Study: Non-Hospitalized and Hospitalized Patients Share Similar Symptom Patterns | | 208 | Only report age in intervals for some groups: 21-30: 8.2%, 31-40: 23.1%, 41-50: 38.9%, 51-60: 19.7%, and 61+:8.2% | 163 (77.9%) | Only refer to "hospitalised", "ICU" and "non-hospitalised" | NR, only report symptoms lasting for >1 month to >1 year |
| 2022 | Patel | Five-item odorant test as an indicator of COVID-19 infection in a general population | | 1042 (50 positive and 992 negative) | Mean 43.22 (SD 16.47, range 18-80; positive), 45.95 (SD 18.125, range 17-92; negative) | 26 (52%; positive), 586 (59.1%; negative) | NR | NR |
| 2021 | Schambeck | Phantosmia, parosmia, and dysgeusia are prolonged and late-onset symptoms of covid-19 | | 44 | Median 41 (range 23-62) | 29 (65.9%) | Asymptomatic, mild, fever, and hospitalised | Acute, otherwise not specified other than "100 days after infection" |
| 2022 | Di Stadio | Olfactory Dysfunction, Headache, and Mental Clouding in Adults with Long-COVID-19: What Is the Link between Cognition and Olfaction? A Cross-Sectional Study | | 152 | Mean 41.2 (SD 11) | 102 (67%) | NR | NR |
| 2021 | Coelho | Quality of life and safety impact of COVID-19 associated smell and taste disturbances | | 322 | Mean 41.57 (SD 13.72, range 18-78) | 258 (80.4%) | NR | NR |
| 2022 | Bhat | "An integrative approach with Ayurveda and Traditional Chinese Acupuncture in post covid parosmia - A case study" | | 1 | 20 | 1 (100%) | NR | Post-COVID |
| 2022 | Damiano | Association between chemosensory impairment with neuropsychiatric morbidity in post-acute COVID-19 syndrome: results from a multidisciplinary cohort study | | 701 | 55.3 (SD 14.6) | 334 (47.6%) | Moderate to severe | Post-acute |
| 2022 | Said | Clinical factors associated with lower health scores in COVID-19-related persistent olfactory dysfunction | | 286 | Mean 37.15 (SD 13.08) | 216 (75.52%) | NR | Post-COVID (although report COVID-19 incidents from <1 month to >12 months ago) |
| 2022 | Fjaeldstad | The Effects of Olfactory Loss and Parosmia on Food and Cooking Habits, Sensory Awareness, and Quality of Life-A Possible Avenue for Regaining Enjoyment of Food | | 688 (of which 166 were controls) | Mean 47 (IQR 34-58) | 417 (80%) | NR | NR |
| 2021 | Polat | Olfactory and Gustatory Dysfunctions in COVID-19 Patients: From a Different Perspective | | 217 | Mean 41.74 (SD 18-76, range 18-76) | 88 (40.6%) | Mild-to-moderate | NR, only report assessing participants during the first symptomatic week and 21 days after diagnosis |
| 2021 | Hosseininasab | Follow-up and outcome of olfactory and gustatory dysfunctions in patients with covid-19 | | 20 | NR | 11 (55%) | Only refer to "hospitalised" and "outpatients" | NR, only report assessing participants at least 2 weeks following symptom onset |
| 2022 | Leung | Phantosmia may predict long-term measurable olfactory dysfunction after COVID-19 | | 100 | Mean 42.2 (SD 15.6) | 55 (55%) | Only refer to "ambulatory" and "hospitalised" | Post-COVID |
| 2022 | Boscolo-Rizzo | COVID-19-Related Quantitative and Qualitative Olfactory and Gustatory Dysfunction: Long-Term Prevalence and Recovery Rate. | | 253 | Median 48 (IQR 38-56) | 158 (62.5%) | Mild-to-moderate | NR, only refer to “long term olfactory impairment” after 24 months after COVID-19 |
| 2023 | Lechien | Platelet-rich plasma injection in the olfactory clefts of COVID-19 patients with long-term olfactory dysfunction | | 87 | Mean 41.6 (SD 14.6) | 62 (71.3%) | NR | Post-COVID |
| 2023 | Lechien | Effectiveness of olfactory training in COVID-19 patients with olfactory dysfunction: a prospective study | | 57 | Mean 40.55 (SD 11.66) | 23 (40%) | NR | Post-COVID |
| 2022 | Lechien | Prevalence and 24-month recovery of olfactory dysfunction in COVID-19 patients: A multicentre prospective study | | 171 | Mean 45 (SD 12) | 120 (70%) | Mild, moderate, severe, critical | NR |
| 2022 | Abo El Naga | The potential therapeutic effect of platelet-rich plasma in the treatment of post-COVID-19 parosmia | | 60 | Mean 28.9 (SD 6.31; case group), 30.07 (SD 5.74; controls) | 19 (63.3%); case group, 21 (70%; controls) | NR, only refer to “outpatients” | Post-COVID |
| 2022 | Schambeck | Two-Year Follow-Up on Chemosensory Dysfunction and Adaptive Immune Response after Infection with SARS-CoV-2 in a Cohort of 44 Healthcare Workers | | 44 | Median 43 (range 24-63) | 29 (65.9%) | NR, only report that no participant was hospitalised | NR, but report that follow-up was done about two years after initial infection |
| 2023 | Tuna | Post-COVID Parosmia in Women May be Associated with Low Estradiol Levels | | 23 | Mean 32 (range 18-45) | 23 (100%) | NR | Post-COVID |
| 2022 | Bousquet | Phantom smells: a prevalent COVID-19 symptom that progressively sets in | | 4691 | Mean 40.4 (SD 12.5) | 3763 (80.2%) | NR | NR |
| 2022 | De Luca | Effect of Ultra-Micronized Palmitoylethanolamide and Luteolin on Olfaction and Memory in Patients with Long COVID: Results of a Longitudinal Study | | 69 | Mean 40.6 (SD 10.5) | 43 (62%) | NR | Post-COVID |
| 2023 | Molnár | The Correlation between the Results of the Sniffin' Sticks Test, Demographic Data, and Questionnaire of Olfactory Disorders in a Hungarian Population after a SARS-CoV-2 Infection | | 110 | Mean 37.5 (SD 11) | 75 (68%) | NR, only refer to “outpatient clinic” | NR |
| 2023 | Bérubé | Olfactory Training Impacts Olfactory Dysfunction Induced by COVID-19: A Pilot Study | | 50 | Mean 44.9 (SD 7.4; intervention group), mean 44.5 (SD 10.1; controls) | 16 (64%; intervention group), 17 (68%; controls) | NR | Post-COVID |
| 2022 | Callejón-Leblic | Analysis of Prevalence and Predictive Factors of Long-Lasting Olfactory and Gustatory Dysfunction in COVID-19 Patients | | 777 | Mean 46.8 (SD 13.9, range 23-89) | 70 (68.6%) | Mild, moderate, severe | Post-COVID |
| 2022 | Silverberg | Predictors of chronic COVID-19 symptoms in a community-based cohort of adults | | 372 | Median 42 (range 18-76) | 178 (47.9%) | NR, only refer to “ambulatory cohort” | Post-COVID |
| 2023 | Hunter | Proof-of-concept: SCENTinel 1.1 rapidly discriminates COVID-19-related olfactory disorders | | 381 | Mean NR (range 18-75) | NR | NR | NR |
| 2022 | Lechien | Injection of Platelet Rich Plasma in the Olfactory Cleft for COVID-19 Patients With Persistent Olfactory Dysfunction: Description of the Technique | | 1 | 22 | 1 (100%) | NR | Post-COVD |
| 2023 | Khan | Efficacy of Combined Visual-Olfactory Training With Patient-Preferred Scents as Treatment for Patients With COVID-19 Resultant Olfactory Loss: A Randomized Clinical Trial | | 275 | Mean 41 (SD 12) | 236 (86%) | NR | NR, but report symptoms lasting for more than three months |
| 2022 | Lee | Development and Psychometric Validation of the Olfactory Dysfunction Outcomes Rating | | 283 | Mean 47 (SD 14.4) | 198 (73%) | NR | NR |
| 2022 | Overdevest | Chemosensory deficits are best predictor of serologic response among individuals infected with SARS-CoV-2 | | 306 | Median 39 (IQR 32-50) | 196 (64.1%) | NR | NR, but report eligibility criteria of minimum two weeks following infection |
| 2023 | Sekine | Assessment of postviral qualitative olfactory dysfunction using the short SSParoT in patients with and without parosmia | | 63 | Mean 41 (IQR 21.5, range 18-80) | 45 (71%) | NR | Post-COVID |
| 2022 | Moideen | Bilateral Olfactory Bulb Atrophy in Post-COVID-19 Parosmia | | 1 | 68 | 1 (100%) | NR | Post-COVID |
| 2022 | Stankevice | Long-Term COVID-19 Smell and Taste Disorders Differ Significantly from Other Post-Infectious Cases | | 328 | Mean 39.9 (CI 36.7-43; simple post-COVID group), mean 44.5 (CI 41.7-47.2; complex post-COVID group), mean 59.2 (CI 57.1-61.4; non-COVID group) | (74.7%; simple post-COVID group), (70.3%; complex post-COVID group), (67.1%; non-COVID group) | NR | Post-COVID |
| 2022 | Schwab | Recovery rates and parosmia in olfactory loss during the COVID-19 era | | 733 | Median 43 (IQR 31-52) | 544 (74.2%) | NR | Post-COVID |
| 2023 | Fjaeldstad | Olfactory training in long COVID-19 patients with lasting symptoms including olfactory dysfunction | | 52 | Median 47 (range 15-71) | (71.1%) | NR | Post-COVID |
| 2022 | Turk | What Is the Long-Term Findings of Olfactory and Taste Loss due to COVID-19? | | 77 | Mean 42 (SD 14.6) | 41 (53.2%) | Mild-moderate and severe-critical | NR |
| 2022 | Menzel | Parosmia as a predictor of a better olfactory function in COVID-19: a multicentric longitudinal study for upper respiratory tract infections | | 147 | Mean 44.3 (SD 15.5, range 18-83) | 96 (65.3%) | NR | NR |
| 2023 | Pendolino | Long-term quality-of-life impairment in patients with more than 1-year COVID-19-related olfactory dysfunction | | 60 | Mean 44.4 (SD 12.3; normosmia group), mean 43 (SD 13.1; dysosmia group) | 7 (50%; normosmia group), 33 (71.7%; dysosmia group) | Mild to moderate | Post-COVID |
| 2022 | McWilliams | Recovery from Covid-19 smell loss: Two-years of follow up | | 946 | Mean 43.8 (SD 13.9, range 18-82) | 753 (79.6%) | NR | Post-COVID |
|  | | | | | | | | |
| NA: Not applicable, NR: Not reported, C-19: COVID-19 | | | | | | | | |
| Sample size is reported for total number of included participants, unless otherwise specified | | | | | | | | |
| Age is reported in mean/median and standard deviation (SD), inter-quartile range (IQR), confidence intervals (CI), and range | | | | | | | | |
| Sex/gender is reported in number of females in number and percentage in brackets | | | | | | | | |

## Supplementary Table 2. Individual studies’ design.

| Year | 1^st^ author | Title | Format | Study type | Follow-up | Language | SETTING |
| --- | --- | --- | --- | --- | --- | --- | --- |
| 2021 | Lechien | Epidemiological, otolaryngological, olfactory and gustatory outcomes according to the severity of COVID-19: a study of 2579 patients | Online or consultation (for patients who could not complete questionnaire themselves) | Cross-sectional | NA | NR | 18 European hospitals |
| 2020 | Lechien | Olfactory and gustatory dysfunctions as a clinical presentation of mild-to-moderate forms of the coronavirus disease (COVID-19): a multicenter European study | Consultation/phone/online depending on severity | Cross-sectional | NA | NR | 12 European hospitals |
| 2021 | Saussez | Short-Term Efficacy and Safety of Oral and Nasal Corticosteroids in COVID-19 Patients with Olfactory Dysfunction: A European Multicenter Study | Online questionnaire | Longitudinal | Baseline, 1 and 2 months | NR | 3 European hospitals |
| 2021 | Ninchritz-Becerra | Subjective evaluation of smell and taste dysfunction in patients with mild COVID-19 in Spain | Online questionnaire | Longitudinal | 1 month (recovery was evaluated between 1-4, 5-8, 9-14, and >15 days. | NR | NR |
| 2021 | Lechien | Prevalence and 6-month recovery of olfactory dysfunction: a multicentre study of 1363 COVID-19 patients | Online questionnaire | Cross-sectional | NA | NR | 18 European hospitals |
| 2020 | Lechien | Objective olfactory evaluation of self-reported loss of smell in a case series of 86 COVID-19 patients | Online questionnaire | Cross-sectional | NA | NR | Department of Anatomy, University of Mons, Belgium |
| 2022 | AlHazmi | COVID-19 infection related olfactory dysfunction in Saudi Arabia: Community-based study | Online questionnaire | Cross-sectional | NA | NR | Qassim region, Saudi-Arabia |
| 2022 | Boscolo-Rizzo | Parosmia assessment with structured questions and its functional impact in patients with long-term COVID-19-related olfactory dysfunction | Consultation | Cross-sectional | NA | NR | Trieste University Hospital for smell and taste disorders |
| 2021 | Weiss | Tracking smell loss to identify healthcare workers with SARS-CoV-2 infection | Online questionnaire | Longitudinal | 3 months | English | Yale New Haven Hospital/ Yale School of Medicine, USA |
| 2022 | Otte | Impact of COVID-19-Mediated Olfactory Loss on Quality of Life | NR | Cross-sectional | NA | German | Outpatient clinic, Germany |
| 2020 | Qiu | Olfactory and Gustatory Dysfunction as an Early Identifier of COVID-19 in Adults and Children: An International Multicenter Study | NR | Longitudinal | 3 weeks after baseline | NR | Shanghai, Wuhan and Shenzhen in China, Paris in France and Dresden in Germany |
| 2022 | Prem | Long-lasting olfactory dysfunction in COVID-19 patients | Home-based (per post) and consultation | Longitudinal | Mean 216 days between onset of olfactory dysfunction and follow-up | NR | University of Vienna, Austria |
| 2021 | Liu | Olfactory-related Quality of Life Adjustments in Smell Loss during the Coronavirus-19 Pandemic | Home-based (per post) | Cross-sectional | NA | NR | University of Vienna, Austria |
| 2021 | Chu | Implementing a COVID-19 specialist smell clinic: experience at the Wrightington, Wigan and Leigh Teaching Hospitals (WWL), NHS Foundation Trust, United Kingdom | Consultation | Longitudinal | Baseline, follow-up at 4 months | English | Wrightington, Wigan and Leigh Teaching Hospitals, NHS Foundation Trust, UK |
| 2021 | Karni | Self-Rated Smell Ability Enables Highly Specific Predictors of COVID-19 Status: A Case-Control Study in Israel | Telephone questionnaire | Cross-sectional case-control | NA | Hebrew/Israeli | NR |
| 2021 | Klein | Onset, duration and unresolved symptoms, including smell and taste changes, in mild COVID-19 infection: a cohort study in Israeli patients | Telephone questionnaire | Longitudinal | Baseline, 3 weeks, 6 weeks, and 6 months | Hebrew/Israeli | NR |
| 2020 | Parma | More than smell - COVID-19 is associated with severe impairment of smell, taste, and chemesthesis | Online questionnaire | Cross-sectional | NA | English, French, German, Italian, Japanese, Kannada, Norwegian, Spanish, Swedish, and Turkish | Online global survey |
| 2022 | Ohla | A follow-up on quantitative and qualitative olfactory dysfunction and other symptoms in patients recovering from COVID-19 smell loss | Online questionnaire | Longitudinal | Baseline, follow-up between 2 and 10 months | English, Spanish, Italian, Dutch, and French | Online survey |
| 2022 | Delgado-Losada | Long-Lasting Olfactory Dysfunction in Hospital Workers Due to COVID-19: Prevalence, Clinical Characteristics, and Most Affected Odorants | Online questionnaire | Cross-sectional | NA | Spanish | Hospital Central de la Cruz Roja San José y Santa Adela, Spain |
| 2021 | Bussière | Chemosensory Dysfunctions Induced by COVID-19 Can Persist up to 7 Months: A Study of Over 700 Healthcare Workers | Online questionnaire and video consultation (for CPT assessment) | Cross-sectional | NA | English and French | Institut National de Santé Publique du Quebec |
| 2022 | Vaira | The Effects of Persistent Olfactory and Gustatory Dysfunctions on Quality of Life in Long-COVID-19 Patients | Online questionnaire | Cross-sectional | NA | Italian and English version of questionnaire | Sassari, Italy |
| 2020 | Biadsee | Olfactory and Oral Manifestations of COVID-19: Sex-Related Symptoms-A Potential Pathway to Early Diagnosis | Online questionnaire | Cross-sectional | NA | NR | Online |
| 2021 | Sayin | Taste and Smell Impairment in COVID-19: An AAO-HNS Anosmia Reporting Tool-Based Comparative Study | Telephone questionnaire | Cross-sectional | NA | NR | Bakirköy Dr. Sadi Konuk Teaching and Research Hospital, Istanbul. Turkey |
| 2021 | Chung | Neurosensory rehabilitation and olfactory network recovery in COVID-19-related olfactory dysfunction | NR | Longitudinal | 4 weeks. Not specified whether the questionnaire is given at follow-up | NR | University of Hong Kong |
| 2020 | Chung | Olfactory Dysfunction in Coronavirus Disease 2019 Patients: Observational Cohort Study and Systematic Review | NR | Cross-sectional | NA | NR | Queen Mary Hospital and Pamela Youde Nethersole Eastern Hospital in Hong Kong |
| 2022 | Lerner | Clinical Features of Parosmia Associated With COVID-19 Infection | Online questionnaire | Longitudinal | Follow-up questionnaire at a mean 244.8 days from symptom onset, range 89-369 days | English | Icahn School of Medicine at Mount Sinai, New York, USA |
| 2022 | Pendolino | The Role of Social Media in Improving Patient Recruitment for Research Studies on Persistent Post-Infectious Olfactory Dysfunction | Online questionnaire | Cross-sectional | NA | English | Royal National ENT and Eastman Dental Hospitals, London, and AbScent Facebook page |
| 2022 | Gupta | Efficacy and Safety of Saline Nasal Irrigation Plus Theophylline for Treatment of COVID-19-Related Olfactory Dysfunction: The SCENT2 Phase 2 Randomized Clinical Trial | Online questionnaire | Longitudinal randomised controlled trial | Baseline, 3 weeks, and 6 weeks | English | Washington University School of Medicine in St Louis, Missouri |
| 2021 | Makaronidis | Distorted chemosensory perception and female sex associate with persistent smell and/or taste loss in people with SARS-CoV-2 antibodies: a community based cohort study investigating clinical course and resolution of acute smell and/or taste loss in people with and without SARS-CoV-2 antibodies in London, UK | Online questionnaire | Longitudinal | Baseline, 4-6 weeks | NR. Supplementary questionnaires are in English | University College London, UK |
| 2020 | Gorzkowski | Evolution of Olfactory Disorders in COVID-19 Patients | Telephone interview | Cross-sectional | NA | French and English | University Hospital of Nancy, France |
| 2021 | Ferdenzi | Recovery from COVID-19-Related Olfactory Disorders and Quality of Life: Insights from an Observational Online Study | Online questionnaire | Cross-sectional | NA | French | Lyon Neuro-science Research Center |
| 2022 | Parker | Emerging Pattern of Post-COVID-19 Parosmia and Its Effect on Food Perception | Online questionnaire | Cross-sectional | NA | NR | University of Reading, UK |
| 2021 | Raad | Parosmia in patients with COVID-19 and olfactory dysfunction. | Online questionnaire | Cross-sectional | NA | NR | Masih Daneshvari Hospital, Iran |
| 2022 | Katsarou | The Greek Collaborative Long COVID Study: Non-Hospitalized and Hospitalized Patients Share Similar Symptom Patterns | Online questionnaire | Cross-sectional | NA | Greek (also provides English translation) | Greece |
| 2022 | Patel | Five-item odorant test as an indicator of COVID-19 infection in a general population | Consultation | Cross-sectional | NA | English | State of Louisiana Offica of Public Health in Central Louisiana and nursing home in Florida, USA |
| 2021 | Schambeck | Phantosmia, parosmia, and dysgeusia are prolonged and late-onset symptoms of covid-19 | NR | Longitudinal | Acute phase (not specified) and 100 days after infection | NR | Helios Klinikum Munchen West, Germany |
| 2022 | Di Stadio | Olfactory Dysfunction, Headache, and Mental Clouding in Adults with Long-COVID-19: What Is the Link between Cognition and Olfaction? A Cross-Sectional Study | Consultation | Cross-sectional | NA | NR | COVID-19 Smell Disorders Centers in Italy |
| 2021 | Coelho | Quality of life and safety impact of COVID-19 associated smell and taste disturbances | Online questionnaire | Longitudinal | Baseline, 2 weeks, 1 month, 3 months, and 6 months | English | Virginia Commonwealth University School of Medicine (Smell and Taste Center) |
| 2022 | Bhat | "An integrative approach with Ayurveda and Traditional Chinese Acupuncture in post covid parosmia - A case study" | Consultation | Case study | Reports symptoms up to 6 months, but not clear whether this involved follow-up or retrospective assessment | Indian | Yenepoya Ayurveda Medical College and Hospital, Naringana |
| 2022 | Damiano | Association between chemosensory impairment with neuropsychiatric morbidity in post-acute COVID-19 syndrome: results from a multidisciplinary cohort study | Consultation/interview | Cross-sectional | NA | NR | Hospital das Clinicas da Faculdade de Medicina da Universidad de Sao Paulo, Brazil |
| 2022 | Said | Clinical factors associated with lower health scores in COVID-19-related persistent olfactory dysfunction | Online questionnaire | Cross-sectional | NA | English | University of California, San Diego |
| 2022 | Fjaeldstad | The Effects of Olfactory Loss and Parosmia on Food and Cooking Habits, Sensory Awareness, and Quality of Life-A Possible Avenue for Regaining Enjoyment of Food | Online questionnaire | Cross-sectional | NA | NR | Regional Hospital Gødstrup, Denmark |
| 2021 | Polat | Olfactory and Gustatory Dysfunctions in COVID-19 Patients: From a Different Perspective | Consulation | Longitudinal | During first week of symptoms, and 21 days following diagnosis | NR | Istanbul Medipol University |
| 2021 | Hosseininasab | Follow-up and outcome of olfactory and gustatory dysfunctions in patients with covid-19 | Consulation and/or phone interview | Longitudinal | Baseline, and at discharge or at least 2 weeks after onset of disease/start of treatment | Farsi | Afzalipour Hospital affiliated to Kerman University of Medical Sciences in South-eastern Iran |
| 2022 | Leung | Phantosmia may predict long-term measurable olfactory dysfunction after COVID-19 | NR | Longitudinal | Baseline, 1 month, 1 year | NR. Supplementary questionnaires are in English | Red de Salud UC-CHRISTUSSantiago, Chile |
| 2022 | Boscolo-Rizzo | COVID-19-Related Quantitative and Qualitative Olfactory and Gustatory Dysfunction: Long-Term Prevalence and Recovery Rate | Interview | Longitudinal | Baseline, 12 months, 24 months | NR | Trieste University Hospital, Italy |
| 2023 | Lechien | Platelet-rich plasma injection in the olfactory clefts of COVID-19 patients with long-term olfactory dysfunction | Online questionnaire | Cross-sectional | NA | NR | Ear, Nose and Throat Dour Medical Center, and CHU Saint-Pierre University Hospital, Brussels, Belgium |
| 2023 | Lechien | Effectiveness of olfactory training in COVID-19 patients with olfactory dysfunction: a prospective study | NR | Longitudinal | Baseline, 6, 12, and 18 months | NR | Ear, Nose and Throat Dour Medical Center, CHU Saint-Pierre University Hospital, and EpiCURA hospital of Baudour, Belgium |
| 2022 | Lechien | Prevalence and 24-month recovery of olfactory dysfunction in COVID-19 patients: A multicentre prospective study | Online questionnaire | Longitudinal | Baseline, 6, 12, 18, and 24 moths | NR | Foch Hospital, Paris, France, and CHU Saint-Pierre University Hospital, and EpiCURA hospital of Baudour, Belgium |
| 2022 | Abo El Naga | The potential therapeutic effect of platelet-rich plasma in the treatment of post-COVID-19 parosmia | Consultation | Longitudinal | Baseline, 1 month | NR | Otorhinolaryngology Department, [Removed for blinding] |
| 2022 | Schambeck | Two-Year Follow-Up on Chemosensory Dysfunction and Adaptive Immune Response after Infection with SARS-CoV-2 in a Cohort of 44 Healthcare Workers | NR | Longitudinal | Baseline, 100, 244, and 721 days | NR | Helios Klinikum Munchen West, Germany |
| 2023 | Tuna | Post-COVID Parosmia in Women May be Associated with Low Estradiol Levels | NR | Cross-sectional | NA | NR | Department of Otorhinola-ryngology, Bursa City Hospital, Turkey |
| 2022 | Bousquet | Phantom smells: a prevalent COVID-19 symptom that progressively sets in | Online questionnaire | Cross-sectional | NA | French | Lyon Neuro-science Research Center |
| 2022 | De Luca | Effect of Ultra-Micronized Palmitoylethanolamide and Luteolin on Olfaction and Memory in Patients with Long COVID: Results of a Longitudinal Study | Consultation | Longitudinal | 90 days | NR | Italy |
| 2023 | Molnár | The Correlation between the Results of the Sniffin' Sticks Test, Demographic Data, and Questionnaire of Olfactory Disorders in a Hungarian Population after a SARS-CoV-2 Infection | Consultation | Cross-sectional | NA | Hungarian | Smell and Gustatory Disorders, Department of Otorhino-laryngology and Head and Neck Surgery, Semmel-weis University |
| 2023 | Bérubé | Olfactory Training Impacts Olfactory Dysfunction Induced by COVID-19: A Pilot Stud | Consultation/videoconsultation | Longitudinal | Baseline, 12 weeks | NR | Canada |
| 2022 | Callejón-Leblic | Analysis of Prevalence and Predictive Factors of Long-Lasting Olfactory and Gustatory Dysfunction in COVID-19 Patients | Consultation | Longitudinal | Baseline, 1 year | NR | Spain |
| 2023 | Hunter | Proof-of-concept: SCENTinel 1.1 rapidly discriminates COVID-19-related olfactory disorders | Online questionnaire and self-administered SCENTinel 1.1 test | Cross-sectional | NA | NR | Monell Chemical Senses Center, Philadelphia |
| 2022 | Silverberg | Predictors of chronic COVID-19 symptoms in a community-based cohort of adults. | Online survey | Cross-sectional | NA | NR | 5 US states |
| 2022 | Lechien | Injection of Platelet Rich Plasma in the Olfactory Cleft for COVID-19 Patients With Persistent Olfactory Dysfunction: Description of the Technique | Consultation | Longitudinal case study | Baseline, 3 weeks | French | Department of Otolaryn-gology, Head and Neck Surgery, EpiCURA Hospital, Baudour, Belgium |
| 2023 | Khan | Efficacy of Combined Visual-Olfactory Training With Patient-Preferred Scents as Treatment for Patients With COVID-19 Resultant Olfactory Loss: A Randomized Clinical Trial | Online questionnaire | Longitudinal | Baseline, 3 months | NR | 41 US states |
| 2022 | Lee | Development and Psychometric Validation of the Olfactory Dysfunction Outcomes Rating | Online questionnaire | Diagnostic study | Baseline, 4-12 weeks | NR | Department of Otolaryngology-Head and Neck Surgery, Washington University School of Medicine, St Louis, Missouri |
| 2022 | Overdevest | Chemosensory deficits are best predictor of serologic response among individuals infected with SARS-CoV-2 | Consultation | Cross-sectional | NA | English | NewYork-Presbytarian Hospital, New York, USA |
| 2023 | Sekine | Assessment of postviral qualitative olfactory dysfunction using the short SSParoT in patients with and without parosmia | Consultation | Cross-sectional | NA | NR | Smell and Taste Clinic of the Department of Otorhinola-ryngology of the Technical University of Dresden |
| 2022 | Moideen | Bilateral Olfactory Bulb Atrophy in Post-COVID-19 Parosmia | Consultation | Cross-sectional case study | NA | NR | Iqraa Internatio-nal Hospital and Research Centre, Calicut, Kerala, India |
| 2022 | Stankevice | Long-Term COVID-19 Smell and Taste Disorders Differ Significantly from Other Post-Infectious Cases | Consultation | Cross-sectional | NA | NR. Supplementary questionnaires are in Danish | Smell and Taste Clinic, University Clinic for Flavour, Balance,and Sleep, ENT Department, Goedstrup Hospital, Denmark. |
| 2022 | Schwab | Recovery rates and parosmia in olfactory loss during the COVID-19 era | Online questionnaire | Longitudinal | Baseline, six rounds of follow-up questionnaires at 2-3 month intervals | NR. Supplementary questionnaires are in Danish | Smell and Taste Clinic, University Clinic for Flavour, Balance,and Sleep, ENT Department, Goedstrup Hospital, Denmark. |
| 2023 | Fjaeldstad | Olfactory training in long COVID-19 patients with lasting symptoms including olfactory dysfunction | Consultation | Longitudinal | Baseline, follow-up visits at 3-4 month intervals | NR. Supplementary questionnaires are in Danish | Smell and Taste Clinic, University Clinic for Flavour, Balance,and Sleep, ENT Department, Goedstrup Hospital, Denmark |
| 2022 | Turk | What Is the Long-Term Findings of Olfactory and Taste Loss due to COVID-19? | Consultation | Longitudinal | Baseline, follow up after at least 12 months | NR | Sisli Hamidiye Etfal Training and Research Hospital, Istanbul, Turkey |
| 2022 | Menzel | Parosmia as a predictor of a better olfactory function in COVID-19: a multicentric longitudinal study for upper respiratory tract infections | Consultation | Longitudinal | Baseline, follow-up after a mean of 3.4 months | NR. Information in e-mail: “written in German” | Dresden, Augsburg,Germany, and Trieste, Italy |
| 2023 | Pendolino | Long-term quality-of-life impairment in patients with more than 1-year COVID-19-related olfactory dysfunction | Consultation | Cross-sectional | NA | NR | Royal National ENT & Eastman Dental Hospitals, London, UK |
| 2022 | McWilliams | Recovery from Covid-19 smell loss: Two-years of follow up | Online questionnaire | Longitudinal | Baseline, 14 days, 1, 3 and 6 months follow-up | NR. Supplementary question is in English | Web-based survey |
| NA: Not applicable, NR: Not reported  Format refers to the administration of the tool  Follow-up specifies the frequency and duration of the study, whenever reported  Language refers to the language of the questionnaire used in the current study | | | | | | | |

## Supplementary Table 3. Tool properties presented by first author and tool.

| Year | 1^st^ author | Title | Number of items | Response design | Scoring procedure |
| --- | --- | --- | --- | --- | --- |
| Yale Jiffy | | | | | |
| 2021 | Weiss | Tracking smell loss to identify healthcare workers with SARS-CoV-2 infection | (2) | Yes/No, VAS from 0 to 10 (where 10 indicate "completely different") | NR |
| SCENTinel 1.1 | | | | | |
| 2023 | Hunter | Proof-of-concept: SCENTinel 1.1 rapidly discriminates COVID-19-related olfactory disorders | (4) | Multiple choice, VAS from 0-100 (where 100 is more intense), and VAS from -100 to 100 (where 100 is very pleasant) | Detection and identification were scored as correct or incorrect. Intensity was scored with a cut-off point of 20, and hedonic rating was scored subtracting the imagined odour rating from the test odour rating |
| Sniffin’ Sticks Parosmia Test (SSParoT) | | | | | |
| 2023 | Sekine | Assessment of postviral qualitative olfactory dysfunction using the short SSParoT in patients with and without parosmia | (4) | 9-point scale | Hedonic range is the difference between two scores from -4 to 4. Hedonic direction is the average of the mean rating of the four odour pairs |
| Chemosensory Perception Test (CPT) | | | | | |
| 2021 | Bussière | Chemosensory Dysfunctions Induced by COVID-19 Can Persist up to 7 Months: A Study of Over 700 Healthcare Workers | (3) | S: "Yes", "No weaker", and "No, different | Only state that "We obtained olfactory scores by averaging these ratings." |
| Taste and smell component, National Health and Nutrition Examination Survey (NHANES) | | | | | |
| 2021 | Lechien | Epidemiological, otolaryngological, olfactory and gustatory outcomes according to the severity of COVID-19: a study of 2579 patients | NR. S: 34 (2) | NR. S: Multiple choice | NR |
| 2020 | Lechien | Olfactory and gustatory dysfunctions as a clinical presentation of mild-to-moderate forms of the coronavirus disease (COVID-19): a multicenter European study | NR. S: 34 (2) | NR. S: Multiple choice | NR |
| 2021 | Saussez | Short-Term Efficacy and Safety of Oral and Nasal Corticosteroids in COVID-19 Patients with Olfactory Dysfunction: A European Multicenter Study | NR. S: 34 (2) | NR. S: Multiple choice | NR |
| 2021 | Ninchritz-Becerra | Subjective evaluation of smell and taste dysfunction in patients with mild COVID-19 in Spain | NR. S: 34 (2) | NR. S: Multiple choice | NR |
| 2021 | Lechien | Prevalence and 6-month recovery of olfactory dysfunction: a multicentre study of 1363 COVID-19 patients | NR. S: 34 (2) | NR. S: Multiple choice | NR |
| 2020 | Lechien | Objective olfactory evaluation of self-reported loss of smell in a case series of 86 COVID-19 patients | NR. S: 34 (2) | NR. S: Multiple choice | NR |
| 2022 | AlHazmi | COVID-19 infection related olfactory dysfunction in Saudi Arabia: Community-based study | NR. S: 10 (2) | NR. S: Multiple choice | NR |
| 2023 | Lechien | Platelet-rich plasma injection in the olfactory clefts of COVID-19 patients with long-term olfactory dysfunction | NR | NR | NR |
| 2023 | Lechien | Effectiveness of olfactory training in COVID-19 patients with olfactory dysfunction: a prospective study | NR | NR | NR |
| 2022 | Lechien | Prevalence and 24-month recovery of olfactory dysfunction in COVID-19 patients: A multicentre prospective study | NR | NR | NR |
| Parosmia questionnaire | | | | | |
| 2022 | Boscolo-Rizzo | Parosmia assessment with structured questions and its functional impact in patients with long-term COVID-19-related olfactory dysfunction | (4) | 4-point Likert scale. | Scored 1 to 4, summed and converted to a percentage, termed Score A, according to the following formula: ((Sum-4)/12) X 100) |
| 2021 | Weiss | Tracking smell loss to identify healthcare workers with SARS-CoV-2 infection | (4) | 4-point Likert scale | Scored from 1 to 4. Scores of 14 or less indicate parosmia |
| 2023 | Tuna | Post-COVID Parosmia in Women May be Associated with Low Estradiol Levels | (4) | 4-point Likert scale | Scored from 1 to 4, with a total score of 16 |
| Questionnaire of Olfactory Disorders (QoD) and anglicised version (eODQ) | | | | | |
| 2022 | Otte | Impact of COVID-19-Mediated Olfactory Loss on Quality of Life | 29 (4) | 4-point Likert scale | Report scoring procedure: scored 0-3, in total 12. Higher scores indicate disorder |
| 2020 | Qiu | Olfactory and Gustatory Dysfunction as an Early Identifier of COVID-19 in Adults and Children: An International Multicenter Study | 29 (4) | NR | NR. S: Scored: 0-3, in total 12. Higher scores indicate disorder |
| 2022 | Prem | Long-lasting olfactory dysfunction in COVID-19 patients | 23. Supplementary matieral includes 29 (4) | 4-point Likert scale | "High scores on parosmia questions (4 questions) assume qualitative OD." Higher scores indicate disorder |
| 2021 | Liu | Olfactory-related Quality of Life Adjustments in Smell Loss during the Coronavirus-19 Pandemic | 23 (4) | 4-point Likert scale | "We reversed the coding of scores for further analysis so that (i) higher QOD-PAR scores represented higher parosmia-related symptomatology". In supplementary material scores are given from 0-3, in total 12 |
| 2022 | Gupta | Efficacy and Safety of Saline Nasal Irrigation Plus Theophylline for Treatment of COVID-19-Related Olfactory Dysfunction: The SCENT2 Phase 2 Randomized Clinical Trial | (4) | NR | NR |
| 2023 | Molnár | The Correlation between the Results of the Sniffin' Sticks Test, Demographic Data, and Questionnaire of Olfactory Disorders in a Hungarian Population after a SARS-CoV-2 Infection | (4) | 4-point Likert scale | “…“agree” (1), “agree partly”, “disagree partly”, and “disagree” (4).” |
| 2023 | Bérubé | Olfactory Training Impacts Olfactory Dysfunction Induced by COVID-19: A Pilot Study | NR | NR | NR |
| 2022 | Lechien | Injection of Platelet Rich Plasma in the Olfactory Cleft for COVID-19 Patients With Persistent Olfactory Dysfunction: Description of the Technique | NR | NR | NR |
| 2021 | Chu | Implementing a COVID-19 specialist smell clinic: experience at the Wrightington, Wigan and Leigh Teaching Hospitals (WWL), NHS Foundation Trust, United Kingdom | 32 (2) | 6-point Likert scale | NR. S: 0-100. Higher scores indicate disorder |
| Global Consortium for Chemosensory Research (GCCR) Questionnaire | | | | | |
| 2021 | Karni | Self-Rated Smell Ability Enables Highly Specific Predictors of COVID-19 Status: A Case-Control Study in Israel | NR. S: 47 (1) | NR. S: Check all that apply | NR |
| 2021 | Klein | Onset, duration and unresolved symptoms, including smell and taste changes, in mild COVID-19 infection: a cohort study in Israeli patients | NR. S: 47 (1) | NR. S: Check all that apply | NR |
| 2020 | Parma | More than smell - COVID-19 is associated with severe impairment of smell, taste, and chemesthesis | NR. S: 47 (1) | Check all that apply | NR |
| 2022 | Ohla | A follow-up on quantitative and qualitative olfactory dysfunction and other symptoms in patients recovering from COVID-19 smell loss | NR. S: 47 (1) | NR. S: Check all that apply | NR |
| 2022 | Delgado-Losada | Long-Lasting Olfactory Dysfunction in Hospital Workers Due to COVID-19: Prevalence, Clinical Characteristics, and Most Affected Odorants. | NR. S: 47 (1) | NR. S: Check all that apply | NR |
| Self-developed questionnaire/screening question(s) | | | | | |
| 2022 | Boscolo-Rizzo | Parosmia assessment with structured questions and its functional impact in patients with long-term COVID-19-related olfactory dysfunction | (1) | NR | NR |
| 2021 | Bussière | Chemosensory Dysfunctions Induced by COVID-19 Can Persist up to 7 Months: A Study of Over 700 Healthcare Workers | NR. S: 47 (1). CPT: (3) | NR. S: Check all that apply. CPT: "Yes", "No weaker", and "No, different" | Only state that "We obtained olfactory scores by averaging these ratings." |
| 2022 | Vaira | The Effects of Persistent Olfactory and Gustatory Dysfunctions on Quality of Life in Long-COVID-19 Patients | NR. S: 30 (3) | NR. S: Multiple choice, free text entry | NR |
| 2020 | Biadsee | Olfactory and Oral Manifestations of COVID-19: Sex-Related Symptoms-A Potential Pathway to Early Diagnosis | 31 (1) | Yes/No, describe | Only state that scores were summarised |
| 2021 | Sayin | Taste and Smell Impairment in COVID-19: An AAO-HNS Anosmia Reporting Tool-Based Comparative Study | NR. S: 17 (1) | NR. S: Yes/No, multiple choice | NR |
| 2021 | Chung | Neurosensory rehabilitation and olfactory network recovery in COVID-19-related olfactory dysfunction | NR. S: (2) | NR. S: No/Yes - comments | NR |
| 2020 | Chung | Olfactory Dysfunction in Coronavirus Disease 2019 Patients: Observational Cohort Study and Systematic Review | NR. S: (2) | NR. S: No/Yes - comments | NR |
| 2022 | Lerner | Clinical Features of Parosmia Associated With COVID-19 Infection | NR | NR. Sl: Choose the following that apply, describe, VAS (1-10, where 10 is severe impairment) | NR |
| 2022 | Pendolino | The Role of Social Media in Improving Patient Recruitment for Research Studies on Persistent Post-Infectious Olfactory Dysfunction | NR. S: 18 (2) | NR. S: Yes/No | NR |
| 2021 | Makaronidis | Distorted chemosensory perception and female sex associate with persistent smell and/or taste loss in people with SARS-CoV-2 antibodies: a community based cohort study investigating clinical course and resolution of acute smell and/or taste loss in people with and without SARS-CoV-2 antibodies in London, UK | NR. S: (1) | NR. S: No/Yes, if yes describe (optional) | NR |
| 2020 | Gorzkowski | Evolution of Olfactory Disorders in COVID-19 Patients | 16 (2) | NR. S: Yes/No | NR |
| 2021 | Ferdenzi | Recovery from COVID-19-Related Olfactory Disorders and Quality of Life: Insights from an Observational Online Study | NR. S: 14 (2) | NR. S: Yes/No, "describe" for question about phantosmia | NR |
| 2022 | Parker | Emerging Pattern of Post-COVID-19 Parosmia and Its Effect on Food Perception | NR. S: 61 | Multiple choice and free text entry | NR |
| 2021 | Raad | Parosmia in patients with COVID-19 and olfactory dysfunction | NR. S: (1) | NR. S: Check all that apply | NR |
| 2022 | Katsarou | The Greek Collaborative Long COVID Study: Non-Hospitalized and Hospitalized Patients Share Similar Symptom Patterns | 14 (1) | Free text entry | NR |
| 2022 | Patel | Five-item odorant test as an indicator of COVID-19 infection in a general population | NA | Yes/No | NR |
| 2021 | Schambeck | Phantosmia, parosmia, and dysgeusia are prolonged and late-onset symptoms of covid-19 | NR | Yes/No, describe, multiple choice | NR |
| 2022 | Di Stadio | Olfactory Dysfunction, Headache, and Mental Clouding in Adults with Long-COVID-19: What Is the Link between Cognition and Olfaction? A Cross-Sectional Study | NR. Information in e-mail: (4) | NR. Information in e-mail: Yes/No, VAS (0-10, where 10 is totally altered) | NR |
| 2021 | Coelho | Quality of life and safety impact of COVID-19 associated smell and taste disturbances | NR. Information in e-mail: (1) | Multiple choice | NR |
| 2022 | Bhat | "An integrative approach with Ayurveda and Traditional Chinese Acupuncture in post covid parosmia - A case study" | NA | Multiple choice, VAS (0-10 cm, where 10 is severe) | NR |
| 2022 | Damiano | Association between chemosensory impairment with neuropsychiatric morbidity in post-acute COVID-19 syndrome: results from a multidisciplinary cohort study | (1) | Yes/No. In e-mail: "Select everything applies" | NR |
| 2022 | Said | Clinical factors associated with lower health scores in COVID-19-related persistent olfactory dysfunction | (1) | Yes/No | NR |
| 2022 | Fjaeldstad | The Effects of Olfactory Loss and Parosmia on Food and Cooking Habits, Sensory Awareness, and Quality of Life-A Possible Avenue for Regaining Enjoyment of Food | (2) | Multiple choice | NR |
| 2021 | Polat | Olfactory and Gustatory Dysfunctions in COVID-19 Patients: From a Different Perspective | NA | NA | NR |
| 2021 | Hosseininasab | Follow-up and outcome of olfactory and gustatory dysfunctions in patients with covid-19 | NR | NR. Information in e-mail: "… indicate presence, time of onset and duration…" | NR |
| 2022 | Leung | Phantosmia may predict long-term measurable olfactory dysfunction after COVID-19 | 6 | NR. S: Yes/No, check all that apply | NR |
| 2022 | Boscolo-Rizzo | COVID-19-Related Quantitative and Qualitative Olfactory and Gustatory Dysfunction: Long-Term Prevalence and Recovery Rate | NR | Yes/No | NR |
| 2022 | Abo El Naga | The potential therapeutic effect of platelet-rich plasma in the treatment of post-COVID-19 parosmia | NR | VAS (0-10) | “Reaching 0-1 on the visual analog scale was a complete improvement” |
| 2022 | Schambeck | Two-Year Follow-Up on Chemosensory Dysfunction and Adaptive Immune Response after Infection with SARS-CoV-2 in a Cohort of 44 Healthcare Workers | NR | Yes/No, describe, multiple choice | NR |
| 2022 | Bousquet | Phantom smells: a prevalent COVID-19 symptom that progressively sets in | NR. S: 14 (2) | NR. S: Yes/No, "describe" for question about phantosmia | NR |
| 2022 | De Luca | Effect of Ultra-Micronized Palmitoylethanolamide and Luteolin on Olfaction and Memory in Patients with Long COVID: Results of a Longitudinal Study | 52 | 0 (normal perception) – 10 (extremely distorted smell) | NR |
| 2022 | Callejón-Leblic | Analysis of Prevalence and Predictive Factors of Long-Lasting Olfactory and Gustatory Dysfunction in COVID-19 Patients | NR | Yes/No, VAS (0-100) | NR |
| 2023 | Hunter | Proof-of-concept: SCENTinel 1.1 rapidly discriminates COVID-19-related olfactory disorders | (1) | Check all that apply | NA |
| 2022 | Silverberg | Predictors of chronic COVID-19 symptoms in a community-based cohort of adults | (2) | Check all that apply, free text entry | NA |
| 2022 | Overdevest | Chemosensory deficits are best predictor of serologic response among individuals infected with SARS-CoV-2 | (3) | Check all that apply | NA |
| 2022 | Moideen | Bilateral Olfactory Bulb Atrophy in Post-COVID-19 Parosmia | (1) | NR | NR |
| 2022 | Stankevice | Long-Term COVID-19 Smell and Taste Disorders Differ Significantly from Other Post-Infectious Cases | (2) | Yes (Rarely, often, always), No, Don’t know | NR |
| 2022 | Schwab | Recovery rates and parosmia in olfactory loss during the COVID-19 era | (7) | NR. Information in e-mail: Yes/No/Don’t know, always, often, rarely, never, check all that apply | NR |
| 2023 | Fjaeldstad | Olfactory training in long COVID-19 patients with lasting symptoms including olfactory dysfunction | (7) | NR. Information in e-mail: Yes/No/Don’t know, always, often, rarely, never, check all that apply | NR |
| 2022 | Turk | What Is the Long-Term Findings of Olfactory and Taste Loss due to COVID-19? | (2) | Yes/No (if yes, duration) | NR |
| 2022 | Menzel | Parosmia as a predictor of a better olfactory function in COVID-19: a multicentric longitudinal study for upper respiratory tract infections | (4) | NR. Information in e-mail: 4-point Likert scale | NR. Information in e-mail: “Using this questionnaire all patients underwent a consultation with a medical doctor, where the diagnosis of parosmia and phantosmia were checked regarding the answers of the patients.” |
| 2023 | Pendolino | Long-term quality-of-life impairment in patients with more than 1-year COVID-19-related olfactory dysfunction | (1) | NR | NR |
| 2022 | McWilliams | Recovery from Covid-19 smell loss: Two-years of follow up | (1) | NR. Information in e-mail: Check all that apply | NR |
| Global Clinical Impression Scale (CGI) | | | | | |
| 2022 | Gupta | Efficacy and Safety of Saline Nasal Irrigation Plus Theophylline for Treatment of COVID-19-Related Olfactory Dysfunction: The SCENT2 Phase 2 Randomized Clinical Trial | (1) | 7-point Likert scale | NR |
| Olfactory Dysfunction Outcomes Rating (ODOR) | | | | | |
| 2021 | Gupta | Efficacy and Safety of Saline Nasal Irrigation Plus Theophylline for Treatment of COVID-19-Related Olfactory Dysfunction: The SCENT2 Phase 2 Randomized Clinical Trial | (2) | 5-point Likert scale | NR |
| 2023 | Khan | Efficacy of Combined Visual-Olfactory Training With Patient-Preferred Scents as Treatment for Patients With COVID-19 Resultant Olfactory Loss: A Randomized Clinical Trial | 28 (NR) | Ranging from “no difficulty” and “very rarely bothered” (0 points) to “complete difficulty” and “very frequently bothered” (4 points) | 112 possible points. The higher the score, the greater the severity |
| 2022 | Lee | Development and Psychometric Validation of the Olfactory Dysfunction Outcomes Rating | 28 (NR) | "Very rarely bothered" (0), "Rarely bothered" (1), "Occacionally bothered" (2), "Frequently bothered" (3), "Very frequently bothered" (4) | 112 possible points. The higher the score, the greater the severity |
|  | | | | | |
| Note that each row may contain more than one tool, as some authors have included several tools in the study | | | | | |
| NA: Not applicable, NR: Not reported, S: Reported in supplementary material of the included study | | | | | |
| Number of items are presented in brackets for questions specific to qualitative olfactory dysfunction, otherwise numbers refer to the total number of items in the tool. | | | | | |

## Supplementary Table 4. Item and response formulations in tools presented per author and tool.

| Year | | 1^st^ author | | Title | | Item and response formulations | | |
| --- | --- | --- | --- | --- | --- | --- | --- | --- |
| Yale Jiffy | | | |  | | |  | |
| 2021 | Weiss | | | Tracking smell loss to identify healthcare workers with SARS-CoV-2 infection | | | **"Does it smell different from normal?"**, and **"Please indicate how different it is"** | |
| SCENTinel 1.1 | | | | | | | | |
| 2023 | Hunter | | | Proof-of-concept: SCENTinel 1.1 rapidly discriminates COVID-19-related olfactory disorders | | | NR | |
| Sniffin’ Sticks Parosmia Test (SSParoT) | | | | | | | | |
| 2023 | Sekine | | | Assessment of postviral qualitative olfactory dysfunction using the short SSParoT in patients with and without parosmia | | | NR for SSParoT, but report that “We asked patient if they have either parosmia or phantosmia and we explained each symptom in great detail, for example, “Parosmia typically presents itself as a uniform, unpleasant odor. Odors are perceived different from what they used to smell, e.g., coffee smells like smoke”, “Patients with phantosmia are those who affected smell a pleasant or unpleasant odor even though no odor is present”.” | |
| Chemosensory Perception Test (CPT) | | | | | | |  | |
| 2021 | Bussière | | | Chemosensory Dysfunctions Induced by COVID-19 Can Persist up to 7 Months: A Study of Over 700 Healthcare Workers | | | **"Is the peanut butter smell the same as usual?"**  "Yes", "No weaker", and "No, different" | |
| Taste and Smell Component, National Health and Nutrition Examination Survey (NHANES) | | | | | | | | |
| 2021 | Lechien | | | Epidemiological, otolaryngological, olfactory and gustatory outcomes according to the severity of COVID-19: a study of 2579 patients | | | **"Do some smells bother your altough they do not bother other people"** and **"Do you sometimes smell an unpleasant, bad or burning odor when nothing is there?**  "Yes", "No", "Refused", "Don't know", "Missing" | |
| 2020 | Lechien | | | Olfactory and gustatory dysfunctions as a clinical presentation of mild-to-moderate forms of the coronavirus disease (COVID-19): a multicenter European study | | |  |  |
| 2021 | Saussez | | | Short-Term Efficacy and Safety of Oral and Nasal Corticosteroids in COVID-19 Patients with Olfactory Dysfunction: A European Multicenter Study | | |  |  |
| 2021 | Ninchritz-Becerra | | | Subjective evaluation of smell and taste dysfunction in patients with mild COVID-19 in Spain | | |  |  |
| 2021 | Lechien | | | Prevalence and 6-month recovery of olfactory dysfunction: a multicentre study of 1363 COVID-19 patients. | | |  |  |
| 2020 | Lechien | | | Objective olfactory evaluation of self-reported loss of smell in a case series of 86 COVID-19 patients | | |  |  |
| 2023 | Lechien | | | Platelet-rich plasma injection in the olfactory clefts of COVID-19 patients with long-term olfactory dysfunction | | |  |  |
| 2023 | Lechien | | | Effectiveness of olfactory training in COVID-19 patients with olfactory dysfunction: a prospective study | | |  |  |
| 2022 | Lechien | | | Prevalence and 24-month recovery of olfactory dysfunction in COVID-19 patients: A multicentre prospective study | | |  |  |
| 2022 | AlHazmi | | | COVID-19 infection related olfactory dysfunction in Saudi Arabia: Community-based study | | | Reported in the results section (not specified if wording is the same in questions asked as in reported results): **"Recently, have you had a problem with your sense of smell, with odors not smelling as they should?"**, and **"Recently, have ghost odors appeared (odors that were not actually there, e.g. burning smell, bad smell)?"**  "Yes", "No", "Refused", "Don't know", "Missing" | |
| Parosmia questionnaire | | | | | | |  | |
| 2022 | Boscolo-Rizzo | | | Parosmia assessment with structured questions and its functional impact in patients with long-term COVID-19–related olfactory dysfunction | | | **"Food tastes different than it should because of a problem with odors"**, **"I always have a bad smell in my nose, even if no odor source is present"**, **"Odors that are pleasant to others are unpleasant to me"**, and **"The biggest problem is not that I do not or only weakly perceive odors, but that they smell different than they should"**  "This is always the case", "This is often the case", "This is rarely the case", "This is never the case" | |
| 2021 | Weiss | | | Tracking smell loss to identify healthcare workers with SARS-CoV-2 infection | | |  |  |
| 2023 | Tuna | | | Post-COVID Parosmia in Women May be Associated with Low Estradiol Levels | | |  |  |
| Questionnaire of Olfactory Disorders (QoD) and angliciced version (eODQ) | | | | | | | | |
| 2022 | Otte | | | Impact of COVID-19-Mediated Olfactory Loss on Quality of Life | | | **"Food tastes different than it should because of a problem with odors"**, **"Sometimes I think I can smell something bad, even when other people can't"**, **"Some of the smells that I find unpleasant, other people find pleasant"**, and **"One of my biggest problems is that smells smell different to what they used to before my accident"**  "I agree", "I agree partly", "I disagree partly", "I disagree" | |
| 2020 | Qiu | | | Olfactory and Gustatory Dysfunction as an Early Identifier of COVID-19 in Adults and Children: An International Multicenter Study | | |  |  |
| 2022 | Prem | | | Long-lasting olfactory dysfunction in COVID-19 patients | | |  |  |
| 2021 | Liu | | | Olfactory-related Quality of Life Adjustments in Smell Loss during the Coronavirus-19 Pandemic | | |  |  |
| 2023 | Molnár | | | The Correlation between the Results of the Sniffin' Sticks Test, Demographic Data, and Questionnaire of Olfactory Disorders in a Hungarian Population after a SARS-CoV-2 Infection | | |  |  |
| 2022 | Gupta | | | Efficacy and Safety of Saline Nasal Irrigation Plus Theophylline for Treatment of COVID-19-Related Olfactory Dysfunction: The SCENT2 Phase 2 Randomized Clinical Trial | | | NR | |
| 2023 | Bérubé | | | Olfactory Training Impacts Olfactory Dysfunction Induced by COVID-19: A Pilot Study | | |  |  |
| 2022 | Lechien | | | Injection of Platelet Rich Plasma in the Olfactory Cleft for COVID-19 Patients With Persistent Olfactory Dysfunction: Description of the Technique | | |  |  |
| 2021 | Chu | | | Implementing a COVID-19 specialist smell clinic: experience at the Wrightington, Wigan and Leigh Teaching Hospitals (WWL), NHS Foundation Trust, United Kingdom. | | | **"Often I perceive a bad taste/smell, regardless whether a potential odour/taste is present"**, and **"My biggest problem is not that odours are less intense (or absent), but that things smell different from what they used to"**  "Agree", "Partly agree", "Disagree partly", "Disagree", "Don't understand the question", "I think the question has no value" | |
| Global Consortium for Chemosensory Research (GCCR) Questionnaire | | | | | | | | |
| 2021 | Karni | | | Self-Rated Smell Ability Enables Highly Specific Predictors of COVID-19 Status: A Case-Control Study in Israel | | | **"Since becoming ill, do smells..."**  "Smell weaker than they did before", "Smell different compared to before being ill (i.e. the quality of smell has changed)", "I can smell things that aren't there (e.g. I can smell random burning when nothing is on fire)", "Without any changes", "Other" | |
| 2021 | Klein | | | Onset, duration and unresolved symptoms, including smell and taste changes, in mild COVID-19 infection: a cohort study in Israeli patients | | |  |  |
| 2020 | Parma | | | More than smell - COVID-19 is associated with severe impairment of smell, taste, and chemesthesis | | | **"Have you experienced any of the following changes in smell...?"**  "I cannot smell at all/Smells smell less strong than they did before", "Smells smell different than they did before (the quality of smell has changed", "I can smell things that are'nt there (e.g. I can smell burning when nothing is on fire)", "Sense of smell fluctuates (e.g. comes and goes)" | |
| 2022 | Ohla | | | A follow-up on quantitative and qualitative olfactory dysfunction and other symptoms in patients recovering from COVID-19 smell loss | | |  |  |
| 2022 | Delgado-Losada | | | Long-Lasting Olfactory Dysfunction in Hospital Workers Due to COVID-19: Prevalence, Clinical Characteristics, and Most Affected Odorants | | |  |  |
| Self-developed questionnaire in full version | | | | | | |  | |
| 2021 | Bussière | | | Chemosensory Dysfunctions Induced by COVID-19 Can Persist up to 7 Months: A Study of Over 700 Healthcare Workers | | | **"Are you experiencing any of the following changes in smell with your recent respiratory illness diagnosis?"**  "I cannot smell at all / Smells smell less strong than they did before", "Smells smell different than they did before (the quality of smell has changed)", "I can smell things that aren't there (for example I smell burning when nothing is on fire)", "Sense of smell fluctuates (comes and goes)", "No changes noticed", "Other (Specify)" | |
| 2022 | Vaira | | | The Effects of Persistent Olfactory and Gustatory Dysfunctions on Quality of Life in Long-COVID-19 Patients | | | **"Were there changes to your sense of smell during the infection?"**  "Yes, things smelled different than I expected them to (distortions, or parosmia)", "Yes, I smelled things that other people couldn’t smell (smell phantoms, or phantosmia)",  **"How is your smell now?"**  "Things smell different than I expect them to (distortions, or parosmia)", "I smell things that other people can't smell (smell phantoms, or phantosmia)" | |
| 2020 | Biadsee | | | Olfactory and Oral Manifestations of COVID-19: Sex-Related Symptoms-A Potential Pathway to Early Diagnosis | | | **"Is your perception of smells distorted since the onset of the infection, if yes, describe"** | |
| 2021 | Sayin | | | Taste and Smell Impairment in COVID-19: An AAO-HNS Anosmia Reporting Tool-Based Comparative Study | | | **"Definition of smell impairment (if present)"**  "Anosmia", "Hyposmia", "Parosmia" | |
| 2021 | Chung | | | Neurosensory rehabilitation and olfactory network recovery in COVID-19-related olfactory dysfunction | | | **"Smell history"**  "Anosmia", "Hyposmia", "Parosmia", "Cacosmia" | |
| 2020 | Chung | | | Olfactory Dysfunction in Coronavirus Disease 2019 Patients: Observational Cohort Study and Systematic Review | | |  |  |
| 2022 | Lerner | | | Clinical Features of Parosmia Associated With COVID-19 Infection | | | Baseline questionnaire: **"When you first noticed a change in your smell, did you experience a total loss of smell, decrease in smell or distortion of smell?"**  Follow-up questionnaire: **"Parosmia is defined as a change in the normal perception of smells, such as when the smell of something familiar is distorted, or when something that normally smells pleasant now smells foul"**. **"... did you experience smell distortion with any of the following?"**  “phantom smells (perception of a smell in the absence of that object)”, “burning smell”, “rotten meat or flesh smell”, “gasoline smell”, “cigarette smoke smell”, “chemical smell”, and “other”, followed by “please describe” | |
| 2022 | Pendolino | | | The Role of Social Media in Improving Patient Recruitment for Research Studies on Persistent Post-Infectious Olfactory Dysfunction | | | **"Do you have parosmia (distorted sense of smell)?"**, and **"Do you have phantosmia (smelling things that have no obvious origin)?"**  "Yes", "No" | |
| 2022 | Gupta | | | Efficacy and Safety of Saline Nasal Irrigation Plus Theophylline for Treatment of COVID-19-Related Olfactory Dysfunction: The SCENT2 Phase 2 Randomized Clinical Trial | | | Question about presence: NR | |
| 2021 | Makaronidis | | | Distorted chemosensory perception and female sex associate with persistent smell and/or taste loss in people with SARS-CoV-2 antibodies: a community based cohort study investigating clinical course and resolution of acute smell and/or taste loss in people with and without SARS-CoV-2 antibodies in London, UK | | | **"Have you noticed that odours smell different than they used to?"**  "No", "Yes", "If yes, describe (optional)" | |
| 2020 | Gorzkowski | | | Evolution of Olfactory Disorders in COVID-19 Patients | | | **"Have you ever perceived an odor that did not correspond to olfactory stimulation? (For example, coffee that smells like burnt rubber)"**, and **"Have you ever perceived a smell without any olfactory stimulation?"**  “Yes”, “No” | |
| 2021 | Ferdenzi | | | Recovery from COVID-19-Related Olfactory Disorders and Quality of Life: Insights from an Observational Online Study | | | **"In the past few days/weeks, have smells seemed different from what they usually are (i.e., they don't smell the same)?"**, and **"In the past few days/weeks, have you had any olfactory hallucinations (phantom smells)?"**  "Yes", "No", "describe" (only for the latter question) | |
| 2022 | Bousquet | | | Phantom smells: a prevalent COVID-19 symptom that progressively sets in | | | **"In the past few days/weeks, have you had any olfactory hallucinations (phantom smells)?"**  "Yes", "No", "describe" | |
| 2022 | Parker | | | Emerging Pattern of Post-COVID-19 Parosmia and Its Effect on Food Perception | | | **"Are you currently experiencing smell distortion (parosmia; where things smell different to they did before)?"**  "Yes", "No"  **“When did you start experiencing smell distortions (parosmia)?” -** "Date: ... “  **"What does … smell like to you now?"**  "… smells like it did before", "…smells distorted. Please use 2-3 words to describe …" , "I can’t smell coffee at all", and "I am not familiar with or don’t eat coffee so cannot answer this question"  **“Do you like the smell of ... now?”**  “… smells pleasant”, “… smells neither pleasant nor unpleasant”, “… smells unpleasant now, but I used to like it”, “… has always smelt unpleasant”, and “The smell of ... is so bad I feel as if I want to gag/vomit/leave the room”. For bathroom smells:  **"Describe how ... smells to you now"**  "... no longer smells unpleasant", "... smells just as unpleasant as before"  **"Please list any all other items which currently have a distorted smell."** | |
| 2021 | Raad | | | Parosmia in patients with COVID-19 and olfactory dysfunction | | | **"Which of the following best describes the change in your sense of smell?"**  "I feel that my sense of smell is weaker than before, meaning that I can still smell but with lower intensity than I used to", "I have completely lost my sense of smell", "My sense of smell has changed, meaning that I perceive an abnormal, often unpleasant, smell", "I constantly perceive an unpleasant smell without any source of odour around me" | |
| 2022 | Katsarou | | | The Greek Collaborative Long COVID Study: Non-Hospitalized and Hospitalized Patients Share Similar Symptom Patterns | | | **"Long COVID symptoms (please use a comma to separate each symptom)"** | |
| 2022 | Leung | | | Phantosmia may predict long-term measurable olfactory dysfunction after COVID-19 | | | **“Do you feel that some things smell different from what they previously used to smell? (e.g. coffee used to smell one way, and now it smells another way, commonly unpleasant)”** "Yes", "No"  **“The characteristics that describe these distorted smells are: Frequency”**  “They occur on a daily basis”, “They are infrequent”  **“Impact”**  “They are unpleasant”, “You have lost weight due to reduced food ingestion because of these unpleasant odors.”, “You have not lost weight”  **“Intensity”**  “They are very intense”, “They are mild”  **“How are these symptoms evolved in time?”**  “Diminished”, “Increased”, “No change”  **“Do you perceive smells that are not really present, or that are not perceived by other people when asked?”**  “Yes”, “No”  **“The characteristics that describe these distorted smells are: Frequency”**  “They occur on a daily basis”, “They are infrequent”  **“Impact”**  “They are unpleasant”, “You have lost weight due to reduced food ingestion because of these unpleasant odors.”, “You have not lost weight”  **“Intensity”**  “They are very intense”, “They are mild”  **“How are these symptoms evolved in time?”**  “Diminished”, “Increased”, “No change” | |
| 2023 | Hunter | | | Proof-of-concept: SCENTinel 1.1 rapidly discriminates COVID-19-related olfactory disorders | | | **“Thinking about TODAY, are you experiencing any of the following? Please check all that apply.”**  “I cannot smell anything (anosmia)”, “I can smell odors but they are weaker than usual (hyposmia)”, “I can smell strong odors, but they smell differently than they typically smell (parosmia)”, “I smell things that aren’t there (e.g. smoke smell when there is no fire, phantosmia)”, “Other”, “No” | |
| 2022 | Overdevest | | | Chemosensory deficits are best predictor of serologic response among individuals infected with SARS-CoV-2 | | | **“In the 2 weeks during your coronavirus symptoms your SMELL was (select all that apply):”**  “Normal, my baseline/usual”, “Absent, my sense of smell was completely gone”, “Diminished, my sense of smell was less than normal”, “Heightened, my sense of smell was overly sensitive to some or all scents”, “Distorted, my perception of smells was different from normal”, “Odd, I smelled scents or odors that no one else could”  **“Currently, regarding your SMELL, do you notice any of the following?”**  **“**Heightened, my sense of smell is overly sensitive to some or all scents”, “Distorted, my perception of smells is different from normal”, “Odd, I smell scents or odors that no one else can”  **“How often do you notice your SMELL problem?”**  **“**All the time”, “Some of the time, comes and goes” | |
| Self-developed questionnaire as reported | | | | | | |  | |
| 2022 | Boscolo-Rizzo | | | Parosmia assessment with structured questions and its functional impact in patients with long-term COVID-19–related olfactory dysfunction | | | **"Do you smell odors differently compared with previous experiences?"**, and **"Do you smell odors in the absence of an apparent source?"**  "Yes", "No" | |
| 2022 | Boscolo-Rizzo | | | COVID-19-Related Quantitative and Qualitative Olfactory and Gustatory Dysfunction: Long-Term Prevalence and Recovery Rate | | |  |  |
| 2022 | Patel | | | Five-item odorant test as an indicator of COVID-19 infection in a general population | | | **"Does anything smell different in the last year?"**  "Yes", "No" | |
| 2021 | Schambeck | | | Phantosmia, parosmia, and dysgeusia are prolonged and late-onset symptoms of covid-19 | | | **“Were certain smells changed?**  "No", "Yes"  **Describe which smells were changed and how"**,  **“Did you smell anything that wasn’t there? (Did you hallucinate smell(s)?)"**  "No", "Yes"  **"Name the hallucinated smell(s) and describe their experience of phantosmia"**  "Pleasant", "Unpleasant" and "Known", "Unknown"  **"Current olfactory function"**  "As before", "Changed", "Hallucinatory", "Diminished" | |
| 2022 | Schambeck | | | Two-Year Follow-Up on Chemosensory Dysfunction and Adaptive Immune Response after Infection with SARS-CoV-2 in a Cohort of 44 Healthcare Workers | | |  |  |
| 2022 | Di Stadio | | | Olfactory Dysfunction, Headache, and Mental Clouding in Adults with Long-COVID-19: What Is the Link between Cognition and Olfaction? A Cross-Sectional Study | | | NR. Information in e-mail: **"Did you perceive the odors differently after COVID-19 infection? If yes: did you smell bad odors? ie trash."**, **"Did you perceive distorted odors?"**, **"Only in case of parosmia: Could you score how much is altered the odor with a number from 0 (normal) to 10 (it is totally altered/ unrecognizable)"** | |
| 2021 | Coelho | | | Quality of life and safety impact of COVID-19 associated smell and taste disturbances | | | NR. Information in e-mail: **"Did you experience the following:"** "Abnormal smells (things smell different than expected)", "phantom smells (I smell things that are not there)" | |
| 2022 | Bhat | | | An integrative approach with Ayurveda and Traditional Chinese Acupuncture in post covid parosmia - A case study | | | NR. Information in e-mail: "The patient was asked to indicate discomfortness of her symptom on a 10 cm VAS where 10 was "parosmia" and 0 was "normal". The severity of parosmia was measured by asking **"To what extent does this symptom hamper your day to day activity?"**, with the following alternatives: normal, mild alteration, moderate alteration and severe alteration." | |
| 2022 | Damiano | | | Association between chemosensory impairment with neuropsychiatric morbidity in post-acute COVID-19 syndrome: results from a multidisciplinary cohort study | | | NR. Information in e-mail: **"You had any of the following changes in smell/smell after respiratory disease by COVID-19? (Select everything applies)"**  "I can't smell anything / The smells are less strong than that were before”, “Smells are different from than before (the quality of the smell changed)”, “I smell things that who are not close (e.g. example, I smell burned when nothing is on fire)”, “Smell fluctuates (goes and comes)”, “I had no changes" | |
| 2022 | Said | | | Clinical factors associated with lower health scores in COVID-19-related persistent olfactory dysfunction | | | **"Do you currently have an altered sense of smell due to COVID-19 (aka parosmia)?"**  "Yes", "No" | |
| 2022 | Fjaeldstad | | | The Effects of Olfactory Loss and Parosmia on Food and Cooking Habits, Sensory Awareness, and Quality of Life-A Possible Avenue for Regaining Enjoyment of Food | | | **"Do you have any distortions to your sense of smell (parosmia)?"**  "No", "Yes, I sometimes experience distorted smells (parosmia, altered perception of existing smells)", "Yes, I often experience distorted smells (parosmia), "Yes, I always experience distorted smells (parosmia)  **"Do you experience being able to smell things that are not present (phantosmia)?"**  Same structure, but "…experience phantom smells (phantosmia, smells that are not present)." | |
| 2021 | Polat | | | Olfactory and Gustatory Dysfunctions in COVID-19 Patients: From a Different Perspective | | | **"How would you evaluate your ability to identify odors or taste compared to nonCOVID period of your life?"** | |
| 2021 | Hosseininasab | | | Follow-up and outcome of olfactory and gustatory dysfunctions in patients with COVID-19 | | | NR. Information in e-mail: "Before starting the interview, olfactory dysfunction was defined as a loss, reduction, or change in the sense of smell. The participant was asked to indicate the onset, duration, and time of resolution of smell loss, parosmia, and phantosmia." | |
| 2022 | Abo El Naga | | | The potential therapeutic effect of platelet-rich plasma in the treatment of post-COVID-19 parosmia | | | NR | |
| 2022 | De Luca | | | Effect of Ultra-Micronized Palmitoylethanolamide and Luteolin on Olfaction and Memory in Patients with Long COVID: Results of a Longitudinal Study | | | NR | |
| 2023 | Bérubé | | | Olfactory Training Impacts Olfactory Dysfunction Induced by COVID-19: A Pilot Study | | | “…we asked participants if … they suffered from parosmia”  “Yes”, “No” | |
| 2022 | Callejón-Leblic | | | Analysis of Prevalence and Predictive Factors of Long-Lasting Olfactory and Gustatory Dysfunction in COVID-19 Patients | | | **“Would you say that things smell either different or unpleasant to you now compared to before the infection?”**  “Yes”, “No” | |
| 2022 | Silverberg | | | Predictors of chronic COVID-19 symptoms in a community-based cohort of adult | | | **“Do you still have any lingering symptoms of COVID-19 more than a month after diagnosis?”**  “Change in smell” (amongst a list of symptoms)  **“How long have you had these lingering symptoms (in months)?”** | |
| 2022 | Moideen | | | Bilateral Olfactory Bulb Atrophy in Post-COVID-19 Parosmia | | | NR. Information in e-mail: **"Do you smell odors differently compared with previous experience?"** | |
| 2022 | Turk | | | What Is the Long-Term Findings of Olfactory and Taste Loss due to COVID-19? | | | **“Have you after the COVID-19 disease, experienced a distorted sense of smell?”**  “Yes”, “No”  **“After the diagnosis of COVID-19 disease, have you experienced cacosmia (detection of normal smell as unpleasant or foul smell)?**  “Yes”, “No” | |
| 2022 | Menzel | | | Parosmia as a predictor of a better olfactory function in COVID-19: a multicentric longitudinal study for upper respiratory tract infections | | | **“Because of my odor distortion, food tastes different than it should”**  **“I always have a bad smell in my nose, regardless of whether there is an odor source present”**  **“Many smells that are pleasant for people are unpleasant for me”**  **“The biggest problem is not that I don't notice smells or notice them faintly, but that they smell differently than they should”**  "Completely true", "Mostly true", "Rather not true" or "Not true" (written in German) | |
| 2023 | Pendolino | | | Long-term quality-of-life impairment in patients with more than 1-year COVID-19-related olfactory dysfunction | | | NR. Information in e-mail: **“Are you experiencing any parosmia/phantosmia?”** | |
| 2022 | McWilliams | | | Recovery from Covid-19 smell loss: Two-years of follow up | | | NR. Information in e-mail: **“What is the current status of your sense of smell (check all that apply)?”**  “I can not smell anything”, “I can only smell very strong odors”, “Odors are distorted (for example, a rose does not smell like a rose)”, “I get odor sensations when there are no odors present”, “None of the above” | |
| 2022* | Stankevice | | | Long-Term COVID-19 Smell and Taste Disorders Differ Significantly from Other Post-Infectious Cases | | | **“Are you experiencing odours as distorted?”**  “Yes (rarely/often/always)”, “No”, “Don’t know”  **“Are you experiencing odours that are not present?”**  “Yes (rarely/often/always)”, “No”, “Don’t know” | |
| 2022* | Schwab | | | Recovery rates and parosmia in olfactory loss during the COVID-19 era | | | **“Are you experiencing odours that are distorted?”**  “Yes”, “No”, “Don’t know”  **“Are you experiencing an altered perception of different odours (parosmia = distortion of existing odours)?**  “No”, “Yes, but not daily AND not intensely AND it has no consequences”, “Yes, daily”, “Yes, intensely”, “Yes, it has substantial consequences (for example reduced enjoyment of eating, weight loss, affecting mood)”  **“How many percent of odours in daily life are distorted?”**  **“Do you experience odours that are not present?”**  “Yes”, “No”, “Don’t know”  **“Are you experiencing an altered perception of odours when no odours are present (phantosmia = experience of odours without odours present)?”**  “No”, “Yes, but not daily AND not intensely AND it has no consequences”, “Yes, daily”, “Yes, intensely”, “Yes, it has substantial consequences (for example reduced enjoyment of eating, weight loss, affecting mood)”  **“Food tastes differently than it should because of the odour distortions”**  “Always”, “Often”, “Rarely”, “Never”  **“I have a bad odour in my nose – even when there are no odour sources in my surroundings”**  “Always”, “Often”, “Rarely”, “Never” | |
| 2023* | Fjaeldstad | | | Olfactory training in long COVID-19 patients with lasting symptoms including olfactory dysfunction | | |  |  |
| Clinical Global Impression Scale (CGI) | | | | | | |  | |
| 2022 | Gupta | | | Efficacy and Safety of Saline Nasal Irrigation Plus Theophylline for Treatment of COVID-19-Related Olfactory Dysfunction: The SCENT2 Phase 2 Randomized Clinical Trial | | | **"How intrusive is parosmia or phantosmia in your life now after [3,6] weeks of treatment?"**  "Much better", "Somewhat better", "Slightly better", "Neither better nor worse", "Slightly worse", "Somewhat worse", "Much worse" | |
| Olfactory Dysfunction Outcomes Rating (ODOR) | | | | | | |  | |
| 2022 | Gupta | | | Efficacy and Safety of Saline Nasal Irrigation Plus Theophylline for Treatment of COVID-19-Related Olfactory Dysfunction: The SCENT2 Phase 2 Randomized Clinical Trial | | | **"Due to your sense of smell, how often are you bothered by the following?"**, **"Smells that no one else seems to notice"**, and **"Scents that smell different from before"**  "Very rarely bothered", "Rarely bothered", "Occasionally bothered", "Frequently bothered", "Very frequently bothered" | |
| 2022 | Lee | | | Development and Psychometric Validation of the Olfactory Dysfunction Outcomes Rating | | |  |  |
| 2023 | Khan | | | Efficacy of Combined Visual-Olfactory Training With Patient-Preferred Scents as Treatment for Patients With COVID-19 Resultant Olfactory Loss: A Randomized Clinical Trial | | | NR, only report “no difficulty”, “very rarely bothered”, “complete difficulty”, and “very frequently bothered” | |
|  |  | | |  | | |  | |
| Item and response formulations are presented as items/questions in bold, followed by response alternatives  NR: Not reported, * English translation provided by the authors of this review, as supplementary material provided by e-mail was in Danish | | | | | | | | |
|  | | |  | |  | | |  |

## References

Abo El Naga, H. A., El Zaiat, R. S. & Hamdan, A. M. (2022). The potential therapeutic effect of platelet-rich plasma in the treatment of post-COVID-19 parosmia. *The Egyptian Journal of Otolaryngology,* 38**,** 130.

Alhazmi, W. A., Alodheilah, A. A., Aldharman, S. S., Alandijani, H. A., Alhati, M. M., Alsalmi, G. M., Alahmadi, R. N. & Alnafessah, S. M. (2022). COVID-19 infection related olfactory dysfunction in Saudi Arabia: Community-based study. *MEDICAL SCIENCE,* 26.

American Academy of Otolaryngology – Head and Neck Surgery. (n. d.). *COVID-19 Anosmia Reporting Tool for Clinicians* [Online]. Available: https://www.entnet.org/news/covid-19-anosmia-reporting-tool-initial-findings/ [Accessed 18.01.23].

Bérubé, S., Demers, C., Bussière, N., Cloutier, F., Pek, V., Chen, A., Bolduc-Bégin, J. & Frasnelli, J. (2023). Olfactory Training Impacts Olfactory Dysfunction Induced by COVID-19: A Pilot Study. *ORL,* 85**,** 57-66.

Bhat, A. K., Krishna Kumar, V. & Johnson, J. D. (2022). "An integrative approach with Ayurveda and Traditional Chinese Acupuncture in post covid parosmia - A case study". *Journal of Ayurveda and Integrative Medicine***,** 100560.

Biadsee, A., Biadsee, A., Kassem, F., Dagan, O., Masarwa, S. & Ormianer, Z. (2020). Olfactory and Oral Manifestations of COVID-19: Sex-Related Symptoms-A Potential Pathway to Early Diagnosis. *Otolaryngol Head Neck Surg,* 163**,** 722-728.

Boscolo-Rizzo, P., Hopkins, C., Menini, A., Dibattista, M., Cancellieri, E., Gardenal, N., Tofanelli, M., Valentinotti, R., Lechien, J. R., Vaira, L. A. & Tirelli, G. (2022a). Parosmia assessment with structured questions and its functional impact in patients with long-term COVID-19-related olfactory dysfunction. *INTERNATIONAL FORUM OF ALLERGY & RHINOLOGY*.

Boscolo-Rizzo, P., Tofanelli, M., Zanelli, E., Gardenal, N. & Tirelli, G. (2022b). COVID-19-Related Quantitative and Qualitative Olfactory and Gustatory Dysfunction: Long-Term Prevalence and Recovery Rate. *ORL,* 85**,** 67-71.

Bousquet, C., Bouchoucha, K., Bensafi, M. & Ferdenzi, C. (2022). Phantom smells: a prevalent COVID-19 symptom that progressively sets in. *European Archives of Oto-Rhino-Laryngology*.

Bussiere, N., Mei, J., Levesque-Boissonneault, C., Blais, M., Carazo, S., Gros-Louis, F., De Serres, G., Dupre, N. & Frasnelli, J. (2021). Chemosensory Dysfunctions Induced by COVID-19 Can Persist up to 7 Months: A Study of Over 700 Healthcare Workers. *CHEMICAL SENSES,* 46.

Butowt, R., Bilinska, K. & Von Bartheld, C. S. (2022). Olfactory dysfunction in COVID-19: new insights into the underlying mechanisms. *Trends in Neurosciences,* 46.

Callejón-Leblic, M. A., Martín-Jiménez, D. I., Moreno-Luna, R., Palacios-Garcia, J. M., Alvarez-Cendrero, M., Vizcarra-Melgar, J. A., Fernandez-Velez, C., Reyes-Tejero, I. M., Maza-Solano, J., Gonzalez-Garcia, J., Tena-García, B., Acosta-Mosquera, M. E., Del Cuvillo, A. & Sánchez-Gómez, S. (2022). Analysis of Prevalence and Predictive Factors of Long-Lasting Olfactory and Gustatory Dysfunction in COVID-19 Patients. *Life (Basel),* 12.

Centers for Disease Control and Prevention. (n. d.). *National Healh and Nutrition Examination Survey NHANES 2011-2012* [Online]. National Center for Health Statistics. Available: https://wwwn.cdc.gov/nchs/nhanes/continuousnhanes/default.aspx?BeginYear=2011 [Accessed 17.01.23 2023].

Chu, M., Gopikrishna, D., Rocke, J. & Kumar, B. N. (2021). Implementing a COVID-19 specialist smell clinic: experience at the Wrightington, Wigan and Leigh Teaching Hospitals (WWL), NHS Foundation Trust, United Kingdom. *The Medical journal of Malaysia,* 76**,** 9-13.

Chung, T. W.-H., Sridhar, S., Zhang, A. J., Chan, K.-H., Li, H.-L., Wong, F. K.-C., Ng, M.-Y., Tsang, R. K.-Y., Lee, A. C.-Y., Fan, Z., Ho, R. S.-L., Luk, S. Y., Kan, W.-K., Lam, S. H.-Y., Wu, A. K.-L., Leung, S.-M., Chan, W.-M., Ng, P. Y., To, K. K.-W., Cheng, V. C.-C., Lung, K.-C., Hung, I. F.-N. & Yuen, K.-Y. (2020). Olfactory Dysfunction in Coronavirus Disease 2019 Patients: Observational Cohort Study and Systematic Review. *Open forum infectious diseases,* 7**,** ofaa199-ofaa199.

Chung, T. W.-H., Zhang, H., Wong, F. K. C., Sridhar, S., Chan, K. H., Cheng, V. C. C., Yuen, K. Y., Hung, I. F. N. & Mak, H. K. F. (2021). Neurosensory Rehabilitation and Olfactory Network Recovery in Covid-19-related Olfactory Dysfunction. *BRAIN SCIENCES,* 11.

Coelho, D. H., Reiter, E. R., Budd, S. G., Shin, Y., Kons, Z. A. & Costanzo, R. M. (2021). Quality of life and safety impact of COVID-19 associated smell and taste disturbances. *American journal of otolaryngology,* 42**,** 103001.

Croy, I., Nordin, S. & Hummel, T. (2014). Olfactory disorders and quality of life--an updated review. *Chem Senses,* 39**,** 185-94.

Damiano, R. F., Neto, D. B., Oliveira, J. V. R., Magalhaes Santos, J., Alves, J. V. R., Guedes, B. F., Nitrini, R., De Araujo, A. L., Oliveira, M., Brunoni, A. R., Voegels, R. L., Bento, R. F., Busatto, G., Miguel, E. C., Forlenza, O. V., De Rezende Pinna, F., Utiyama, E. M., Segurado, A. C., Perondi, B., Miethke-Morais, A., Montal, A. C., Harima, L., Fusco, S. R. G., Silva, M. F., Rocha, M. C., Marcilio, I., Rios, I. C., Kawano, F. Y. O., De Jesus, M. A., Kallas, E. G., Carmo, C., Tanaka, C., De Souza, H. P., Marchini, J. F. M., Carvalho, C. R., Ferreira, J. C., Levin, A. S., Oliveira, M. S., Guimaraes, T., Dos Santos Lazari, C., Da Silva Duarte, A. J., Sabino, E., Magri, M. M. C., Barros-Filho, T. E. P., Francisco, M. C. P. B. & Costa, S. F. (2022). Association between chemosensory impairment with neuropsychiatric morbidity in post-acute COVID-19 syndrome: results from a multidisciplinary cohort study. *European Archives of Psychiatry and Clinical Neuroscience*.

De Luca, P., Camaioni, A., Marra, P., Salzano, G., Carriere, G., Ricciardi, L., Pucci, R., Montemurro, N., Brenner, M. J. & Di Stadio, A. (2022). Effect of Ultra-Micronized Palmitoylethanolamide and Luteolin on Olfaction and Memory in Patients with Long COVID: Results of a Longitudinal Study. *Cells,* 11**,** 2552.

Di Stadio, A., Brenner, M. J., De Luca, P., Albanese, M., D'ascanio, L., Ralli, M., Roccamatisi, D., Cingolani, C., Vitelli, F., Camaioni, A., Di Girolamo, S. & Bernitsas, E. (2022). Olfactory Dysfunction, Headache, and Mental Clouding in Adults with Long-COVID-19: What Is the Link between Cognition and Olfaction? A Cross-Sectional Study. *Brain sciences,* 12.

Erskine, S. E. & Philpott, C. M. (2020). An unmet need: Patients with smell and taste disorders. *Clinical Otolaryngology,* 45**,** 197-203.

Ferdenzi, C., Bousquet, C., Aguera, P.-E., Dantec, M., Daudé, C., Fornoni, L., Fournel, A., Kassan, A., Mantel, M., Moranges, M., Moussy, E., Richard Ortegón, S., Rouby, C. & Bensafi, M. (2021). Recovery From COVID-19-Related Olfactory Disorders and Quality of Life: Insights From an Observational Online Study. *Chemical senses,* 46.

Fjaeldstad, A. W., Ovesen, T., Stankevice, D. & Ovesen, T. (2023). Olfactory training in long COVID-19 patients with lasting symptoms including olfactory dysfunction. *Dan Med J,* 70.

Fjaeldstad, A. W. & Smith, B. (2022). The Effects of Olfactory Loss and Parosmia on Food and Cooking Habits, Sensory Awareness, and Quality of Life-A Possible Avenue for Regaining Enjoyment of Food. *FOODS,* 11.

Frasnelli, J. & Hummel, T. (2005). Olfactory dysfunction and daily life. *Eur Arch Otorhinolaryngol,* 262**,** 231-5.

Gary, J. B., Gallagher, L., Joseph, P. V., Reed, D., Gudis, D. A. & Overdevest, J. B. (2023). Qualitative Olfactory Dysfunction and COVID-19: An Evidence-Based Review with Recommendations for the Clinician. *Am J Rhinol Allergy,* 37, 95-101.

Gorzkowski, V., Bevilacqua, S., Charmillon, A., Jankowski, R., Gallet, P., Rumeau, C. & Nguyen, D. T. (2020). Evolution of Olfactory Disorders inCOVID-19 Patients. *LARYNGOSCOPE,* 130**,** 2667-2673.

Gupta, S., Kallogjeri, D., Farrell, N. F., Lee, J. J., Smith, H. J., Khan, A. M. & Piccirillo, J. F. (2022a). Development and Validation of a Novel At-home Smell Assessment. *JAMA OTOLARYNGOLOGY-HEAD & NECK SURGERY,* 148**,** 252-258.

Gupta, S., Lee, J. J., Perrin, A., Khan, A., Smith, H. J., Farrell, N., Kallogjeri, D. & Piccirillo, J. F. (2022b). Efficacy and Safety of Saline Nasal Irrigation Plus Theophylline for Treatment of COVID-19-Related Olfactory Dysfunction: The SCENT2 Phase 2 Randomized Clinical Trial. *JAMA Otolaryngology - Head and Neck Surgery*.

Hosseininasab, A., Farokhnia, M., Arabi Mianroodi, A.-A., Iranmanesh, E., Mohammadi, S., Soltani, A., Ilaghi, M., Shahdforush, S. & Hashemi, A. (2021). Follow-up and Outcome of Olfactory and Gustatory Dysfunctionsin Patients with COVID-19. *Journal of Kerman University of Medical Sciences,* 28**,** 167-172.

Hummel, T., Sekinger, B., Wolf, S. R., Pauli, E. & Kobal, G. (1997). 'Sniffin' sticks': olfactory performance assessed by the combined testing of odor identification, odor discrimination and olfactory threshold. *Chem Senses,* 22**,** 39-52.

Hunter, S. R., Hannum, M. E., Pellegrino, R., O’leary, M. A., Rawson, N. E., Reed, D. R., Dalton, P. H. & Parma, V. (2023). Proof-of-concept: SCENTinel 1.1 rapidly discriminates COVID-19-related olfactory disorders. *Chemical Senses,* 48.

Karni, N., Klein, H., Asseo, K., Benjamini, Y., Israel, S., Nammary, M., Olshtain-Pops, K., Nir-Paz, R., Hershko, A., Muszkat, M. & Niv, M. Y. (2021). Self-Rated Smell Ability Enables Highly Specific Predictors of COVID-19 Status: A Case-Control Study in Israel. *OPEN FORUM INFECTIOUS DISEASES,* 8.

Katsarou, M. S., Iasonidou, E., Osarogue, A., Kalafatis, E., Stefanatou, M., Pappa, S., Gatzonis, S., Verentzioti, A., Gounopoulos, P., Demponeras, C., Konstantinidou, E., Drakoulis, N., Asimakos, A., Antonoglou, A., Mavronasou, A., Spetsioti, S., Kotanidou, A. & Katsaounou, P. (2022). The Greek Collaborative Long COVID Study: Non-Hospitalized and Hospitalized Patients Share Similar Symptom Patterns. *JOURNAL OF PERSONALIZED MEDICINE,* 12.

Khan, A. M., Piccirillo, J., Kallogjeri, D. & Piccirillo, J. F. (2023). Efficacy of Combined Visual-Olfactory Training With Patient-Preferred Scents as Treatment for Patients With COVID-19 Resultant Olfactory Loss: A Randomized Clinical Trial. *JAMA Otolaryngology–Head & Neck Surgery,* 149**,** 141-149.

Klein, H., Asseo, K., Karni, N., Benjamini, Y., Nir-Paz, R., Muszkat, M., Israel, S. & Niv, M. Y. (2021). Onset, duration, and persistence of taste and smell changes and other COVID-19 symptoms: longitudinal study in Israeli patients. *Clin Microbiol Infect.*, 27, 769-74.

Kobal, G., Hummel, T., Sekinger, B., Barz, S., Roscher, S. & Wolf, S. (1996). "Sniffin' sticks": screening of olfactory performance. *Rhinology,* 34**,** 222-6.

Landis, B. N., Frasnelli, J., Croy, I. & Hummel, T. (2010). Evaluating the clinical usefulness of structured questions in parosmia assessment. *Laryngoscope,* 120**,** 1707-13.

Langstaff, L., Pradhan, N., Clark, A., Boak, D., Salam, M., Hummel, T. & Philpott, C. M. (2019). Validation of the olfactory disorders questionnaire for English-speaking patients with olfactory disorders. *Clinical Otolaryngology,* 44**,** 715-728.

Lechien, J. R., Chiesa-Estomba, C. M., Beckers, E., Mustin, V., Ducarme, M., Journe, F., Marchant, A., Jouffe, L., Barillari, M. R., Cammaroto, G., Circiu, M. P., Hans, S. & Saussez, S. (2021a). Prevalence and 6-month recovery of olfactory dysfunction: a multicentre study of 1363 COVID-19 patients. *Journal of internal medicine*.

Lechien, J. R., Chiesa-Estomba, C. M., De Siati, D. R., Horoi, M., Le Bon, S. D., Rodriguez, A., Dequanter, D., Blecic, S., El Afia, F., Distinguin, L., Chekkoury-Idrissi, Y., Hans, S., Delgado, I. L., Calvo-Henriquez, C., Lavigne, P., Falanga, C., Barillari, M. R., Cammaroto, G., Khalife, M., Leich, P., Souchay, C., Rossi, C., Journe, F., Hsieh, J., Edjlali, M., Carlier, R., Ris, L., Lovato, A., De Filippis, C., Coppee, F., Fakhry, N., Ayad, T. & Saussez, S. (2020a). Olfactory and gustatory dysfunctions as a clinical presentation of mild-to-moderate forms of the coronavirus disease (COVID-19): a multicenter European study. *EUROPEAN ARCHIVES OF OTO-RHINO-LARYNGOLOGY,* 277**,** 2251-2261.

Lechien, J. R., Chiesa-Estomba, C. M., Vaira, L. A., De Riu, G., Cammaroto, G., Chekkoury-Idrissi, Y., Circiu, M., Distinguin, L., Journe, F., De Terwangne, C., Machayekhi, S., Barillari, M. R., Calvo-Henriquez, C., Hans, S. & Saussez, S. (2021b). Epidemiological, otolaryngological, olfactory and gustatory outcomes according to the severity of COVID-19: a study of 2579 patients. *European archives of oto-rhino-laryngology : official journal of the European Federation of Oto-Rhino-Laryngological Societies (EUFOS) : affiliated with the German Society for Oto-Rhino-Laryngology - Head and Neck Surgery*.

Lechien, J. R., Ducarme, M., Place, S., Chiesa-Estomba, C. M., Khalife, M., De Riu, G., Vaira, L. A., De Terwangne, C., Machayekhi, S., Marchant, A., Journe, F. & Saussez, S. (2020b). Objective Olfactory Findings in Hospitalized Severe COVID-19 Patients. *Pathogens (Basel, Switzerland),* 9.

Lechien, J. R., Le Bon, S. D. & Saussez, S. (2023a). Platelet-rich plasma injection in the olfactory clefts of COVID-19 patients with long-term olfactory dysfunction. *European Archives of Oto-Rhino-Laryngology,* 280**,** 2351-2358.

Lechien, J. R., Vaira, L. A. & Saussez, S. (2023b). Effectiveness of olfactory training in COVID-19 patients with olfactory dysfunction: a prospective study. *European Archives of Oto-Rhino-Laryngology,* 280**,** 1255-1263.

Lechien, J. R., Vaira, L. A. & Saussez, S. (2023c). Prevalence and 24-month recovery of olfactory dysfunction in COVID-19 patients: A multicentre prospective study. *Journal of Internal Medicine,* 293**,** 82-90.

Lee, J. J., Mahadev, A., Kallogjeri, D., Peterson, A. M., Gupta, S., Khan, A. M., Jiramongkolchai, P., Schneider, J. S. & Piccirillo, J. F. (2022). Development and Psychometric Validation of the Olfactory Dysfunction Outcomes Rating. *JAMA Otolaryngology–Head & Neck Surgery,* 148**,** 1132-1138.

Lerner, D. K., Garvey, K. L., Arrighi-Allisan, A. E., Filimonov, A., Filip, P., Shah, J., Tweel, B., Del Signore, A., Schaberg, M., Colley, P., Govindaraj, S. & Iloreta, A. M. (2022). Clinical Features of Parosmia Associated With COVID-19 Infection. *LARYNGOSCOPE,* 132**,** 633-639.

Leung, J.-S., Cordano, V. P., Fuentes-López, E., Lagos, A. E., García-Huidobro, F. G., Aliaga, R., Díaz, L. A., García-Salum, T., Salinas, E., Toro, A., Callejas, C. A., Riquelme, A., Palmer, J. N., Medina, R. A. & González G, C. (2022). Phantosmia May Predict Long-Term Measurable Olfactory Dysfunction After COVID-19. *The Laryngoscope,* 132**,** 2445-2452.

Liu, D. T., Prem, B., Besser, G., Renner, B. & Mueller, C. A. (2022). Olfactory-related Quality of Life Adjustments in Smell Loss during the Coronavirus-19 Pandemic. *AMERICAN JOURNAL OF RHINOLOGY & ALLERGY,* 36**,** 253-260.

Liu, D. T., Welge-Lüssen, A., Besser, G., Mueller, C. A. & Renner, B. (2020). Assessment of odor hedonic perception: the Sniffin’ sticks parosmia test (SSParoT). *Scientific Reports,* 10**,** 18019.

Makaronidis, J., Firman, C., Magee, C. G., Mok, J., Balogun, N., Lechner, M., Carnemolla, A. & Batterham, R. L. (2021). Distorted chemosensory perception and female sex associate with persistent smell and/or taste loss in people with SARS-CoV-2 antibodies: a community based cohort study investigating clinical course and resolution of acute smell and/or taste loss in people with and without SARS-CoV-2 antibodies in London, UK. *BMC infectious diseases,* 21**,** 221.

Moideen, S., Innisai, A. & Uvais, N. A. (2022). Bilateral Olfactory Bulb Atrophy in Post-COVID-19 Parosmia. *Prim Care Companion CNS Disord,* 24.

Ninchritz-Becerra, E., Soriano-Reixach, M. M., Mayo-Yánez, M., Calvo-Henríquez, C., Martínez-Ruiz De Apodaca, P., Saga-Gutiérrez, C., Parente-Arias, P., Villareal, I. M., Viera-Artiles, J., Poletti-Serafini, D., Alobid, I., Ayad, T., Saussez, S., Lechien, J. R. & Chiesa-Estomba, C. M. (2021). [Subjective evaluation of smell and taste dysfunction in patients with mild COVID-19 in Spain]. *Medicina clinica,* 156**,** 61-64.

Otte, M. S., Haehner, A., Bork, M. L., Klussmann, J. P., Luers, J. C. & Hummel, T. (2022). Impact of COVID-19-Mediated Olfactory Loss on Quality of Life. *ORL-JOURNAL FOR OTO-RHINO-LARYNGOLOGY HEAD AND NECK SURGERY*.

Ouzzani, M., Hammady, H., Fedorowicz, Z. & Elmagarmid, A. (2016). Rayyan—a web and mobile app for systematic reviews. *Systematic Reviews,* 5**,** 210.

Overdevest, J. B., Irace, A. L., Mazzanti, V., Oh, E. J., Joseph, P. V., Devanand, D. P., Bitan, Z. C., Hod, E. A., Gudis, D. A. & Chiuzan, C. (2022). Chemosensory deficits are best predictor of serologic response among individuals infected with SARS-CoV-2. *PLoS One,* 17**,** e0274611.

Page, M. J., Mckenzie, J. E., Bossuyt, P. M., Boutron, I., Hoffmann, T. C., Mulrow, C. D., Shamseer, L., Tetzlaff, J. M., Akl, E. A., Brennan, S. E., Chou, R., Glanville, J., Grimshaw, J. M., Hróbjartsson, A., Lalu, M. M., Li, T., Loder, E. W., Mayo-Wilson, E., Mcdonald, S., Mcguinness, L. A., Stewart, L. A., Thomas, J., Tricco, A. C., Welch, V. A., Whiting, P. & Moher, D. (2021). The PRISMA 2020 statement: an updated guideline for reporting systematic reviews. *BMJ,* 372**,** n71.

Parker, J. K., Kelly, C. E. & Gane, S. B. (2022a). Insights into the molecular triggers of parosmia based on gas chromatography olfactometry. *Communications Medicine,* 2**,** 58.

Parker, J. K., Methven, L., Pellegrino, R., Smith, B. C., Gane, S. & Kelly, C. E. (2022b). Emerging Pattern of Post-COVID-19 Parosmia and Its Effect on Food Perception. *FOODS,* 11.

Parma, V., Hannum, M. E., O'leary, M., Pellegrino, R., Rawson, N. E., Reed, D. R. & Dalton, P. H. (2021). SCENTinel 1.0: Development of a Rapid Test to Screen for Smell Loss. *CHEMICAL SENSES,* 46.

Parma, V., Ohla, K., Veldhuizen, M. G., Niv, M. Y., Kelly, C. E., Bakke, A. J., Cooper, K. W., Bouysset, C., Pirastu, N., Dibattista, M., Kaur, R., Liuzza, M. T., Pepino, M. Y., Schopf, V., Pereda-Loth, V., Olsson, S. B., Gerkin, R. C., Dominguez, P. R., Albayay, J., Farruggia, M. C., Bhutani, S., Fjaeldstad, A. W., Kumar, R., Menini, A., Bensafi, M., Sandell, M., Konstantinidis, I., Di Pizio, A., Genovese, F., Ozturk, L., Thomas-Danguin, T., Frasnelli, J., Boesveldt, S., Saatci, O., Saraiva, L. R., Lin, C., Golebiowski, J., Hwang, L. D., Ozdener, M. H., Guardia, M. D., Laudamiel, C., Ritchie, M., Havlicek, J., Pierron, D., Roura, E., Navarro, M., Nolden, A. A., Lim, J., Whitcroft, K. L., Colquitt, L. R., Ferdenzi, C., Brindha, E. V., Altundag, A., Macchi, A., Nunez-Parra, A., Patel, Z. M., Fiorucci, S., Philpott, C. M., Smith, B. C., Lundstrom, J. N., Mucignat, C., Parker, J. K., Van Den Brink, M., Schmuker, M., Fischmeister, F. P. S., Heinbockel, T., Shields, V. D. C., Faraji, F., Santamaria, E., Fredborg, W. E. A., Morini, G., Olofsson, J. K., Jalessi, M., Karni, N., D'errico, A., Alizadeh, R., Pellegrino, R., Meyer, P., Huart, C., Chen, B., Soler, G. M., Alwashahi, M. K., Welge-Lussen, A., Freiherr, J., De Groot, J. H. B., Klein, H., Okamoto, M., Singh, P. B., Hsieh, J. W., Reed, D. R., Hummel, T., Munger, S. D., Hayes, J. E. & Author, G. G. (2020). More Than Smell - COVID-19 Is Associated With Severe Impairment of Smell,Taste, and Chemesthesis. *CHEMICAL SENSES,* 45**,** 609-622.

Patel, R. A., Torabi, S. J., Kasle, D. A. & Manes, R. P. (2022). Five-item odorant test as an indicator of COVID-19 infection in a general population. *AMERICAN JOURNAL OF OTOLARYNGOLOGY,* 43.

Pendolino, A. L., Navaratnam, A. V., Nijim, J., Kelly, C. E., Randhawa, P. S. & Andrews, P. J. (2022). The Role of Social Media in Improving Patient Recruitment for Research Studies on Persistent Post-Infectious Olfactory Dysfunction. *MEDICINA-LITHUANIA,* 58.

Pendolino, A. L., Tan, H. Q. M., Choi, D., Ottaviano, G. & Andrews, P. J. (2023). Long-term quality-of-life impairment in patients with more than 1-year COVID-19–related olfactory dysfunction. *International Forum of Allergy & Rhinology,* 13**,** 160-164.

Polat, B., Yilmaz, N. H., Altin, G., Atakcan, Z. & Mert, A. (2021). Olfactory and Gustatory Dysfunctions in COVID-19 Patients: From a Different Perspective. *JOURNAL OF CRANIOFACIAL SURGERY,* 32**,** 2119-2122.

Prem, B., Liu, D., Besser, G., Sharma, G., Dultinger, L. E., Hofer, S. V., Matiasczyk, M. M., Renner, B. & Mueller, C. A. (2022). Long-lasting olfactory dysfunction in COVID-19 patients. *EUROPEAN ARCHIVES OF OTO-RHINO-LARYNGOLOGY,* 279**,** 3485-3492.

Qiu, C., Cui, C., Hautefort, C., Haehner, A., Zhao, J., Yao, Q., Zeng, H., Nisenbaum, E. J., Liu, L., Zhao, Y., Zhang, D., Levine, C. G., Cejas, I., Dai, Q., Zeng, M., Herman, P., Jourdaine, C., De With, K., Draf, J., Chen, B., Jayaweera, D. T., Denneny, J. C., 3rd, Casiano, R., Yu, H., Eshraghi, A. A., Hummel, T., Liu, X., Shu, Y. & Lu, H. (2020). Olfactory and Gustatory Dysfunction as an Early Identifier of COVID-19 in Adults and Children: An International Multicenter Study. *Otolaryngology--head and neck surgery : official journal of American Academy of Otolaryngology-Head and Neck Surgery,* 163**,** 714-721.

Raad, N., Ghorbani, J., Safavi Naeini, A., Tajik, N. & Karimi-Galougahi, M. (2021). Parosmia in patients with COVID-19 and olfactory dysfunction. *International Forum of Allergy & Rhinology,* 11**,** 1497-1500.

Said, M., Luong, T., Jang, S. S., Davis, M. E., Deconde, A. S. & Yan, C. R. H. (2022). Clinical factors associated with lower health scores in COVID-19-related persistent olfactory dysfunction. *INTERNATIONAL FORUM OF ALLERGY & RHINOLOGY*.

Saussez, S., Vaira, L. A., Chiesa-Estomba, C. M., Le Bon, S. D., Horoi, M., Deiana, G., Petrocelli, M., Boelpaep, P., Salzano, G., Khalife, M., Hans, S., De Riu, G., Hopkins, C. & Lechien, J. R. (2021). Short-Term Efficacy and Safety of Oral and Nasal Corticosteroids in COVID-19 Patients with Olfactory Dysfunction: A European Multicenter Study. *PATHOGENS,* 10.

Sayin, P., Altinay, M., Cinar, A. S. & Ozdemir, H. M. (2021). Taste and Smell Impairment in Critically Ill Patients With COVID-19: An Intensive Care Unit Study. *Ent-Ear Nose & Throat Journal,* 100**,** 174S-179S.

Schambeck, S. E., Crowell, C. S., Wagner, K. I., D'ippolito, E., Burrell, T., Mijocevic, H., Protzer, U., Busch, D. H., Gerhard, M., Poppert, H. & Beyer, H. (2021). Phantosmia, Parosmia, and Dysgeusia Are Prolonged and Late-Onset Symptoms of COVID-19. *JOURNAL OF CLINICAL MEDICINE,* 10.

Schambeck, S. E., Mateyka, L. M., Burrell, T., Graf, N., Brill, I., Stark, T., Protzer, U., Busch, D. H., Gerhard, M., Riehl, H. & Poppert, H. (2022). Two-Year Follow-Up on Chemosensory Dysfunction and Adaptive Immune Response after Infection with SARS-CoV-2 in a Cohort of 44 Healthcare Workers. *Life,* 12**,** 1556.

Schwab, J. & Fjaeldstad, A. W. (2022). Recovery rates and parosmia in olfactory loss during the COVID-19 era. *Danish Medical Journal,* 69**,** 10.

Sekine, R., Menzel, S., Hähner, A., Mori, E. & Hummel, T. (2023). Assessment of postviral qualitative olfactory dysfunction using the short SSParoT in patients with and without parosmia. *Eur Arch Otorhinolaryngol,* 280**,** 469-472.

Silverberg, J. I., Zyskind, I., Naiditch, H., Zimmerman, J., Glatt, A. E., Pinter, A., Theel, E. S., Joyner, M. J., Hill, D. A., Lieberman, M. R., Bigajer, E., Stok, D., Frank, E. & Rosenberg, A. Z. (2022). Predictors of chronic COVID-19 symptoms in a community-based cohort of adults. *PLoS One,* 17**,** e0271310.

Tuna, B. & Tuna, V. (2023). Post-COVID Parosmia in Women May be Associated with Low Estradiol Levels. *Indian Journal of Otolaryngology and Head & Neck Surgery*.

Turk, B., Aybal, A., Salepci, E., Dizdar, S. K. & Turgut, S. (2022). What Is the Long-Term Findings of Olfactory and Taste Loss due to COVID-19? *Sisli Etfal Hastan Tip Bul,* 56**,** 466-472.

Vaira, L. A., Gessa, C., Deiana, G., Salzano, G., Maglitto, F., Lechien, J. R., Saussez, S., Piombino, P., Biglio, A., Biglioli, F., Boscolo-Rizzo, P., Hopkins, C., Parma, V. & De Riu, G. (2022). The Effects of Persistent Olfactory and Gustatory Dysfunctions on Quality of Life in Long-COVID-19 Patients. *Life-Basel,* 12.

von der Brelie, L., Becker, C. & von der Brelie, C. (2020). Parosmia as an Early Symptom of Acute SARS-CoV-2 Infection. *Deutsches Arzteblatt international,* 117**,** 328.

Weiss, J. J., Attuquayefio, T. N., White, E. B., Li, F., Herz, R. S., White, T. L., Campbell, M., Geng, B., Datta, R., Wyllie, A. L., Grubaugh, N. D., Casanovas-Massana, A., Muenker, M. C., Moore, A. J., Handoko, R., Iwasaki, A., Martinello, R. A., Ko, A. I., Small, D. M. & Farhadian, S. F. (2021). Tracking smell loss to identify healthcare workers with SARS-CoV-2 infection. *PloS one,* 16**,** e0248025.
